# Regulation patterns in signaling networks of cancer

**DOI:** 10.1186/1752-0509-4-162

**Published:** 2010-11-26

**Authors:** Gunnar Schramm, Nandakumar Kannabiran, Rainer König

**Affiliations:** 1 Department of Bioinformatics and Functional Genomics, Institute of Pharmacy and Molecular Biotechnology, Bioquant, University of Heidelberg, Im Neuenheimer Feld 267, 69120 Heidelberg, Germany; 2 Department of Theoretical Bioinformatics, German Cancer Research Center (DKFZ), Im Neuenheimer Feld 280, 69120 Heidelberg, Germany

## Abstract

**Background:**

Formation of cellular malignancy results from the disruption of fine tuned signaling homeostasis for proliferation, accompanied by mal-functional signals for differentiation, cell cycle and apoptosis. We wanted to observe central signaling characteristics on a global view of malignant cells which have evolved to selfishness and independence in comparison to their non-malignant counterparts that fulfill well defined tasks in their sample.

**Results:**

We investigated the regulation of signaling networks with twenty microarray datasets from eleven different tumor types and their corresponding non-malignant tissue samples. Proteins were represented by their coding genes and regulatory distances were defined by correlating the gene-regulation between neighboring proteins in the network (high correlation = small distance). In cancer cells we observed shorter pathways, larger extension of the networks, a lower signaling frequency of central proteins and links and a higher information content of the network. Proteins of high signaling frequency were enriched with cancer mutations. These proteins showed motifs of regulatory integration in normal cells which was disrupted in tumor cells.

**Conclusion:**

Our global analysis revealed a distinct formation of signaling-regulation in cancer cells when compared to cells of normal samples. From these cancer-specific regulation patterns novel signaling motifs are proposed.

## Background

Endogenous signal transduction in cancer cells is systematically disturbed to redirect the cellular decisions from differentiation and apoptosis to proliferation and, later, invasion [1]. Cancer cells acquire their malignancy through accumulation of advantageous gene mutations by which the necessary steps to malignancy are obtained [2]. These selfish adaptations to independence can be described as a result from an evolutionary process of diversity and selection [3]. We were interested to observe the resulting cellular signal transduction on a global view. Experimental high throughput methods such as gene expression profiling with microarrays enable investigating the pathogenic function of tumors on a mesoscopic level. Large-scale gene expression profiles were successfully used to predict clinical outcome [4,5] and improved risk estimation [6]. However these studies didn't relate genes and their expression to a functional context. To gain an understanding on a systems view, gene expression can be mapped onto cellular networks. Several studies have been reported that used gene expression data from microarrays to describe specific characteristics of signaling networks in cancer. Discriminative components of a protein-protein interaction network were identified by comparing gene expression patterns of metastatic and non-metastatic tumors in breast cancer and suited as risk markers for metastasis of breast cancer [7]. New genetic mediators for prostate cancer were found with networks that were reversely engineered from gene expression profiles [8]. Besides this, insights into evolutionary principles were gained by the analysis of gene expression profiles. Gene expression differences were used to define phylogenetic relationships of several *Drosophila *species [9] and a molecular clock for primates [10]. Furthermore, the regulation of signaling in yeast was investigated on a global scale to observe regulatory adaptation to the cellular environment. Yeast responded to exogenous signals by shorter regulatory cascades to enable fast signal propagation [11].

**Figure 1:**
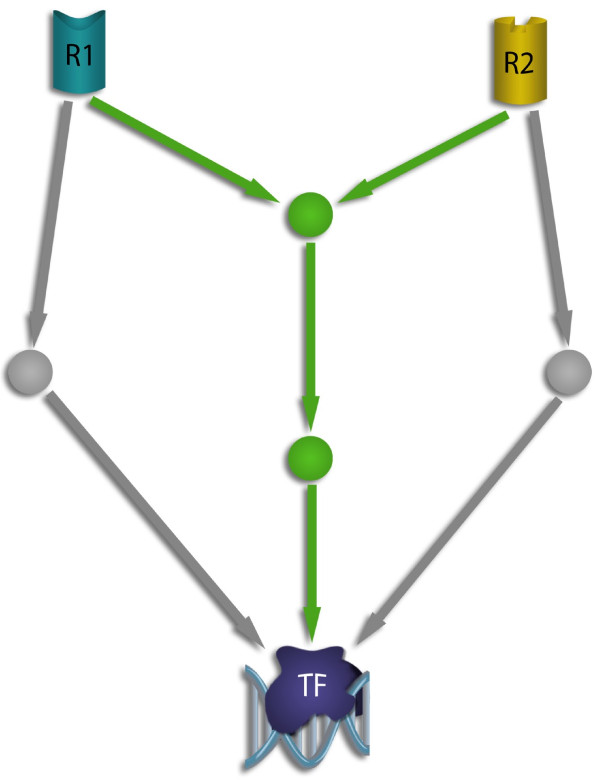
**Comparative cancer motif**. Two different signals are transmitted from two receptors (R1 and R2) to a transcription factor (TF). Green and grey arrows indicate the pathways for normal and cancer cells, respectively. The motif was defined for each pair of pathways (from R1 to TF, and from R2 to TF) such that the pathways of normal cells share at least one common link whereas the pathways for cancer cells did not share any link.

The aim of our work was to detect characteristic signaling properties of cancer cells on a global scale. We compared the regulation of signaling pathways in cancer with normal cells and mapped gene expression data of tumors and their corresponding non-malignant ("normal") samples onto a comprehensive protein-protein-interaction network. For inferring regulation-principles in cellular signal transduction, we used a graph searching algorithm that tracked pathways with the highest correlation in regulation. We investigated twenty tumor-datasets comprising acute myeloid leukemia, esophageal squamos cell-, lung adeno- and renal clear cell carcinoma, breast-, cervical-, head-and-neck-, oral-tongue-, pancreas- and prostate cancer, and vulva interstitial neoplasia. The investigated tumors showed shorter pathways, but a larger extension of the network. The tumors displayed lower frequency of central proteins and links and a higher information entropy (Shannon's information content) in their network. These findings were embedded into a novel signal-regulation motif which was observed considerably more often in normal cells when compared to tumor cells (Figure 1). Similar to the study of Cui and co-workers [12], central proteins (hubs) were enriched with cancer mutations. We observed that these proteins showed higher regulation-integrity in the normal samples whereas the tumor samples showed motifs of regulatory maintenance of the neighbors of hubs.

## Results

### Constructing the signaling networks

**Figure 2:**
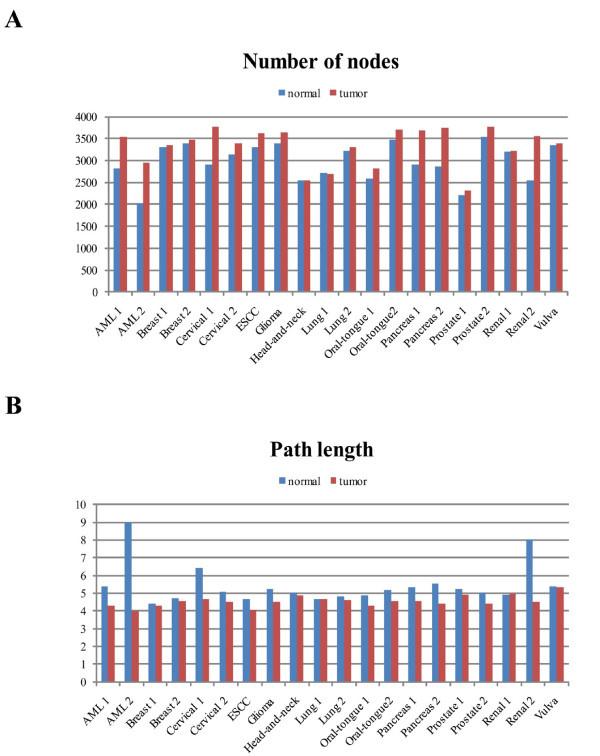
**Network characteristics**. Number of nodes and path lengths for normal and tumor networks of all investigated cancer types. The malignant signaling networks employed a higher number of nodes (average normal: 2973, tumor: 3324, P = 2.3E-03) and were more connected with smaller paths (average normal: 5.4, tumor: 4.6, P = 9.54E-06).

We assembled our signaling network employing a comprehensive data repository of known protein-protein interactions from the literature (HPRD: Human Protein Reference Database [13,14] version 9 from April 13^th^, 2010). Proteins were represented by their coding genes and will also be denoted as nodes of the networks in the following. Gene expression data of each cancer dataset (malignant cells) and the corresponding set of normal samples (non-malignant cells) was mapped onto the nodes of the network. Depending on the coverage of the probes on the microarray chips, the intersection with the HPRD network comprised of 5574 to 8651 nodes including 559 to 706 receptors and 505 to 617 transcription factors (Table 1). Similar to Luscombe and co-workers, we assumed most likely signaling propagation by high co-regulation of genes of two neighboring proteins in the network [11]. We calculated protein-protein-distances for each link (link-distances) by the co-regulation (one minus the absolute value of Pearson's correlation) of the two interacting proteins (Additional file 1: Supplemental Figure S1). The link-distances were higher (lower absolute correlation) in cancer cells compared to normal cells (average of average link-distances in normal: 0.34, and tumor: 0.52, P = 1.53E-05, Table 1). We defined pathways for each pair of receptors (signal-operator) and transcription-factors (signal-receiver) by their shortest paths yielding a range of 282,295 to 435,602 pathways for each of the investigated cancer datasets. The tumor cells showed a distinct higher coverage of the original protein-interaction network for these pathways. Table 1 gives an overview of the network data for the different datasets we analyzed and also the network-coverage of all receptor-transcription-factor pathways for the tumors and the reference samples. From these pathways we constructed specific networks for each tumor and reference sample. For each tumor and normal sample, the constructed networks consisted only of those links and nodes that appeared at least once in their receptor-transcription-factor pathways. Not-appearing links and nodes were discarded (Figure 2 shows the number of nodes in all constructed networks of normal and cancer tissues). We were interested if these networks were specific for the respective tumor type. For this, we extracted all somatically mutated genes for specific cancer tissues from a database (COSMIC [15]) and tested if our tumor networks contained genes which have been described specifically for the respective tumors. We performed enrichment tests (Fisher's exact tests) and found that all tumor networks showed a considerably significant enrichment of their corresponding mutated tumor genes (Additional file 1: Supplemental Table S1).

**Table 1:** Network sizes

	Links in the whole network	Nodes in the whole network	Number of receptors	Number of transcription factors	Percentage of used nodes		Percentage of used links		Average distances of two neighbored nodes (*d_xy_*)	
					**normal**	**tumor**	**normal**	**tumor**	**normal**	**tumor**
**AML 1**	29211	7244	653	578	39.1	48.8	55.5	69.7	0.32	0.59
**AML 2**	23181	5574	559	505	36.4	52.7	48.5	72.9	0.07	0.72
**Breast 1**	29211	7244	653	578	45.7	46.2	65.7	66.3	0.57	0.59
**Breast 2**	33722	8651	706	617	39.1	40.1	57.6	59.4	0.47	0.53
**Cervical 1**	33722	8651	706	617	33.7	43.5	47.4	64.9	0.20	0.52
**Cervical 2**	29211	7244	653	578	43.2	46.8	61.0	66.6	0.38	0.54
**ESCC**	29211	7244	653	578	45.6	49.9	65.4	71.5	0.51	0.73
**Glioma**	33722	8651	706	617	39.1	42.0	56.6	62.6	0.36	0.58
**Head and neck**	23181	5574	559	505	45.8	45.7	62.0	62.0	0.38	0.41
**Lung 1**	23181	5574	559	505	48.7	48.4	67.2	67.8	0.47	0.47
**Lung 2**	29211	7244	653	578	44.4	45.6	62.6	65.3	0.43	0.48
**Oral tongue 1**	23181	5574	559	505	46.5	50.5	63.4	70.0	0.40	0.59
**Oral tongue 2**	33722	8651	706	617	40.3	42.9	59.6	63.5	0.39	0.54
**Pancreas 1**	33722	8651	706	617	33.6	42.7	46.4	63.2	0.26	0.53
**Pancreas 2**	33722	8651	706	617	33.2	43.4	46.3	64.3	0.25	0.57
**Prostate 1**	23181	5574	559	505	39.5	41.6	50.0	53.7	0.28	0.34
**Prostate 2**	33722	8651	706	617	41.0	43.6	60.6	64.9	0.41	0.57
**Renal 1**	29211	7244	653	578	44.1	44.4	63.9	64.3	0.41	0.42
**Renal 2**	33722	8651	706	617	29.5	41.2	40.2	61.0	0.07	0.54
**Vulva**	33722	8651	706	617	38.8	39.2	57.8	58.2	0.34	0.36

### Tumors use shorter paths, more links and less hubs

We calculated a variety of different network-features to characterize specific differences in signaling-regulation of tumor cells and non-malignant cells. The results are given in Table 2 and Table 3 and will be explained in the following. For getting a reasonable estimate of the general tendency of tumors, we calculated the average out of all datasets for cancer and normal networks and performed a significance test of the pair-wise differences between tumor and normal (paired, non-parametric, Wilcoxon-rank test).

**Table 2:** Statistics of general network features

	Number of links		Exponent α		Link frequency		Node frequency		Path length		Increase of path length	
	**normal**	**tumor**	**normal**	**tumor**	**normal**	**tumor**	**normal**	**tumor**	**normal**	**tumor**	**normal**	**tumor**
**AML 1**	16224	20367	1.24	1.28	200	101	720	461	5.40	4.32	1.67	1.57
**AML 2**	11245	16896	0.98	1.30	719	82	1256	384	9.02	3.99	2.04	1.55
**Breast 1**	19188	19357	1.27	1.28	113	108	502	487	4.40	4.32	1.59	1.62
**Breast 2**	19408	20031	1.22	1.23	147	134	605	573	4.70	4.57	1.65	1.63
**Cervical 1**	15983	21877	1.12	1.26	330	121	962	537	6.41	4.64	1.65	1.58
**Cervical 2**	17833	19463	1.23	1.27	152	112	613	500	5.09	4.49	1.54	1.58
**ESCC**	19107	20887	1.25	1.29	122	89	534	424	4.67	4.06	1.56	1.53
**Glioma**	19102	21114	1.18	1.27	172	120	673	543	5.23	4.53	1.55	1.56
**Head and neck**	14365	14364	1.23	1.26	141	136	554	539	5.01	4.87	1.59	1.58
**Lung 1**	15568	15722	1.24	1.28	116	116	484	489	4.65	4.67	1.62	1.58
**Lung 2**	18295	19080	1.24	1.25	135	121	563	525	4.80	4.60	1.59	1.54
**Oral tongue 1**	14698	16223	1.23	1.27	136	94	533	432	4.89	4.31	1.61	1.55
**Oral tongue 2**	20097	21406	1.22	1.25	163	123	649	534	5.19	4.55	1.62	1.62
**Pancreas 1**	15661	21307	1.18	1.25	230	123	798	536	5.32	4.55	1.65	1.60
**Pancreas 2**	15604	21671	1.17	1.25	256	113	841	509	5.54	4.39	1.63	1.59
**Prostate 1**	11590	12450	1.15	1.19	184	154	672	600	5.25	4.93	1.56	1.58
**Prostate 2**	20446	21899	1.23	1.27	156	114	615	510	5.01	4.42	1.72	1.62
**Renal 1**	18660	18791	1.25	1.26	148	144	582	581	4.93	4.95	1.70	1.66
**Renal 2**	13557	20567	1.02	1.26	702	126	1371	552	8.02	4.52	1.55	1.59
**Vulva**	19491	19612	1.22	1.22	192	185	700	688	5.39	5.36	1.72	1.73

**Tendency for tumor**	Up		Up		Down		Down		Down		Down	
**Significance (P-value)**	3.81E-06		1.91E-06		3.81E-06		5.72E-06		5.37E-06		0.021	

**Table 3:** Statistics of topology features and network motifs

	Comparative network motif		Integration motif		Maintenance motif		Cluster coefficient		Cluster coefficient > 0		Information entropy	
	**normal**	**tumor**	**normal**	**tumor**	**normal**	**tumor**	**normal**	**tumor**	**normal**	**tumor**	**normal**	**tumor**
**AML 1**	3.94E+07	1.08E+07	541	27	6	26	0.126	0.113	1924	2344	10.94	12.35
**AML 2**	3.35E+07	3.35E+06	888	0	0	139	0.142	0.119	1373	1971	10.05	12.22
**Breast 1**	1.82E+07	1.88E+07	34	55	10	6	0.116	0.115	2234	2244	12.07	12.11
**Breast 2**	2.62E+07	2.39E+07	272	169	17	15	0.119	0.119	2271	2338	11.98	12.05
**Cervical 1**	5.04E+07	1.19E+07	614	49	4	52	0.134	0.117	1924	2538	10.89	12.12
**Cervical 2**	2.57E+07	1.74E+07	170	58	2	17	0.118	0.116	2081	2278	11.71	12.16
**ESCC**	2.52E+07	1.52E+07	70	8	0	6	0.117	0.112	2236	2435	11.99	12.55
**Glioma**	3.02E+07	2.28E+07	250	92	3	43	0.118	0.115	2242	2450	11.91	12.20
**Head and neck**	1.31E+07	1.24E+07	205	143	3	14	0.129	0.123	1712	1700	11.38	11.39
**Lung 1**	1.42E+07	1.40E+07	83	78	15	18	0.124	0.123	1813	1832	11.82	11.57
**Lung 2**	2.31E+07	1.88E+07	154	99	8	17	0.117	0.117	2137	2233	11.81	12.05
**Oral tongue 1**	1.84E+07	9.83E+06	231	68	1	32	0.130	0.121	1768	1912	11.42	12.04
**Oral tongue 2**	3.06E+07	2.15E+07	144	34	3	30	0.121	0.115	2349	2489	11.76	12.23
**Pancreas 1**	4.20E+07	1.37E+07	1427	115	5	79	0.123	0.114	1873	2457	11.23	12.21
**Pancreas 2**	4.60E+07	1.16E+07	1250	81	0	81	0.126	0.112	1866	2504	11.19	12.33
**Prostate 1**	1.37E+07	8.91E+06	573	398	0	3	0.130	0.126	1407	1485	11.06	11.30
**Prostate 2**	3.07E+07	2.29E+07	178	56	13	24	0.116	0.117	2357	2541	11.79	12.15
**Renal 1**	2.25E+07	2.24E+07	105	123	20	29	0.119	0.119	2181	2190	11.70	11.69
**Renal 2**	5.72E+07	7.05E+06	1227	116	8	116	0.138	0.118	1650	2398	10.56	12.16
**Vulva**	2.81E+07	2.67E+07	231	308	33	23	0.123	0.123	2299	2295	11.57	11.58

**Tendency for tumor**	Down		Down		Up		Down		Up		Up	
**Significance (P-value)**	9.54E-06		5.53E-04		6.34E-04		1.05E-04		1.91E-05		8.20E-05	

**Figure 3:**
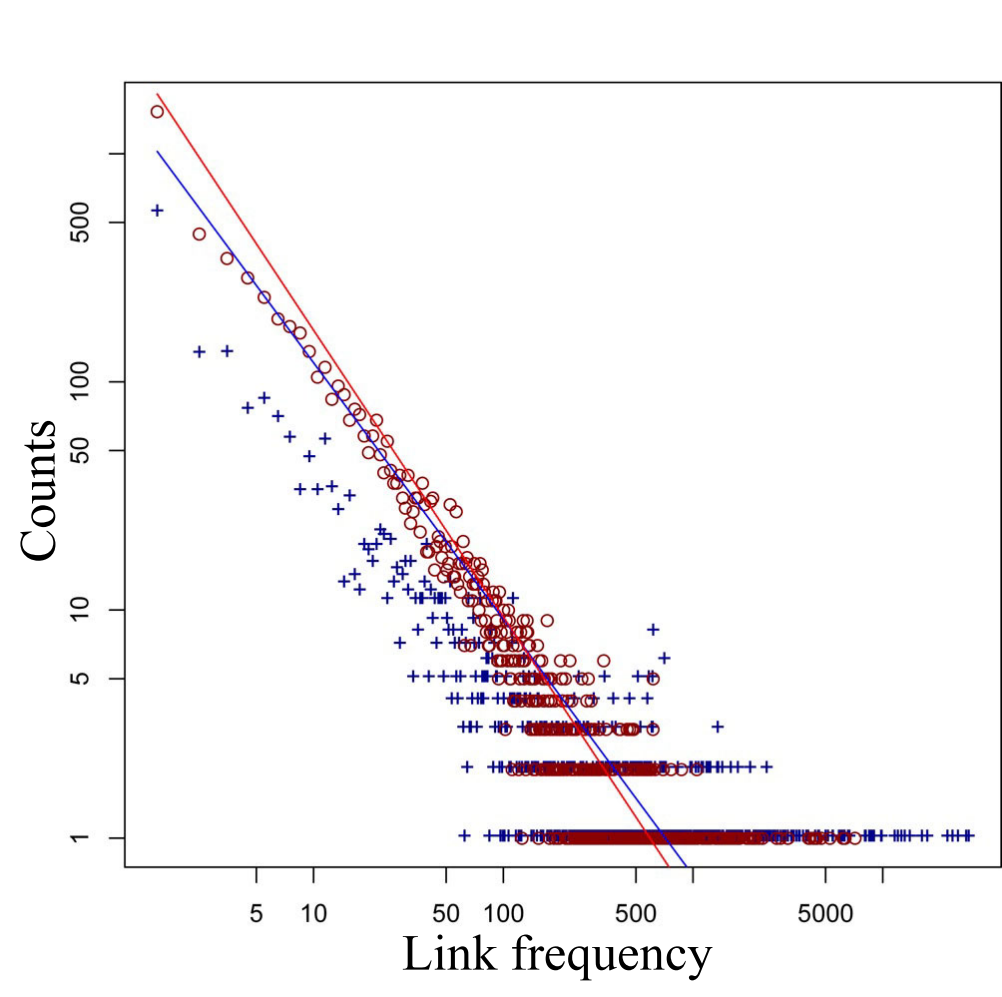
**Link frequency distribution**. The frequency distribution of the links for the network of cervical cancer one (red circles) is shown, in comparison to the distribution of the corresponding normal network (blue crosses). Both networks (tumor and normal) showed typical scale-free distributions. In comparison to the network for normal samples, the cancer network had considerably less hubs and showed a steeper decline of the frequency (higher exponent *α *of the regression function).

The average path-length of cancer networks was less than for non-malignant (average for cancer: 4.58, and normal: 5.50, P = 3.82E-05). We wanted to know how often the same links (interactions) were used for different signaling pathways. For this, we defined the frequency of a link (link-frequency) as the number of receptor-transcription-factor pathways it was involved in. The average link-frequency was obtained by the number of links used in each single pathway from each respective receptor to each transcription factor, divided by the number of all used links. The average link frequency was higher in normal cells (average of average link-frequency for cancer: 122.6, and normal: 234.4, P = 1.53E-05). Similarly, the node frequency was calculated and showed the same tendency (average for cancer: 524.3, and normal: 723.4, P = 2.29E-05). Hence networks of normal cells used more often the same central proteins and interactions for different signaling tasks. Such a hub-like structure is the central characteristic of scale free networks [16]. We were interested if the networks for cancer and normal samples followed these characteristics and if there were distribution differences between them. In deed, the link-frequency distribution of the networks of both entities followed a power law (probability to draw a link with frequency *f *is proportional to *f^-α ^*and *α > 1*). In comparison to the networks from normal cells, the distributions of tumors showed a steeper decline. We calculated the exponent *α *of the distribution and observed larger exponents for cancer networks (P = 1.91E-06). (exemplarily, Figure 3 shows the distributions and the regression function for cervical cancer 1, the distributions for all datasets are given in Additional file 1: Supplemental Figure S2). This agrees with the lower average of their link-frequency. These distributions also show that proteins of high connectivity (hubs) in the networks of normal cells are more abundant (Additional file 1: Supplemental Figure S3 shows some illustrations of networks). The clustering coefficient has been employed as a measure of connectedness of networks [16]. We calculated the clustering coefficient and obtained lower values for the network of cancer cells supporting our findings that cancer showed a tendency for less centralized, less hub-dependent formation (average of cancer: 0.118, and normal: 0.125, P = 4.20E-04). Specifically, the number of nodes with a clustering coefficient greater zero was distinctively higher in cancer cells (average for cancer: 2208 and normal: 1956, P = 7.63E-05).

### Frequently involved genes are enriched with cancer mutated genes

Cui and co-workers compiled a selective list of 284 cancer mutated genes which were derived from large scale sequencing and the literature (Supplementary Table S10 in [12]). We compared this list with the 50 most frequently involved nodes (our hubs) of each network and found significant enrichment for 19 out of 20 normal and tumor datasets (Additional file 1: Supplemental Table S2). We then defined gene-lists of cancer mutated hubs for every cancer by intersecting the hubs of our network with the list of cancer mutated genes of Cui *et al*. (Additional file 1: Supplemental Table S3). Interestingly, most of the genes which showed up in the tumor networks were also present in the normal networks. This may indicate that normal cells intrinsically pave the way for their specific evolvement into malignancy.

### Signaling-regulation in cancer is detached at cancer mutated hubs but maintained in their vicinity

**Figure 4:**
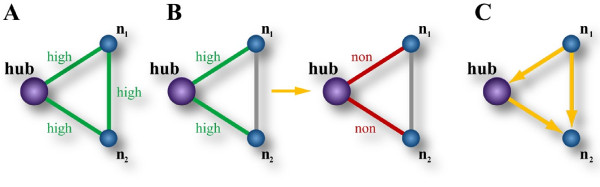
**Triangle motifs**. The motifs were derived for each triple of nodes consisting of a hub and two of its neighbors in the network (n_1_, n_2_) which were also mutually connected. In the integration motif (motif A) all nodes are pair-wise highly co-regulated. Accordingly, the motif is defined by high correlations (low distances) for links hub-n_1_, hub-n_2 _and n_1_-n_2_. In contrast, the maintenance motif (motif B) consisted of a hub which was not co-regulated with its neighbors n_1 _and n_2_. Counted were triangles which pairs of hub-nodes (hub-n_1 _and hub-n_2_) showed high correlation in one tissue type and no correlation in the other, while the correlation of n_1_-n_2 _stayed in the same category. Motif C is a consistent feed-forward loop, taken from the literature [21].

Uri Alon and his co-workers studied occurrences of direction-motifs in triangles and revealed a large variety of substantial characteristics in signaling networks characterized by consistent and non-consistent feed-forward and feedback loops [16]. We were interested in local regulation patterns of the networks at cancer mutated hubs. For this, we analyzed regulation motifs of every triangle consisting of exactly one hub and two of its neighbors which on their part also interact. We defined two regulation motifs. The first motif reflected the degree of regulatory integration of a hub and its network-vicinity and was defined by a high correlation of all pairs of nodes in the triangle motif (integrated motif, motif A in Figure 4). We found this motif significantly more often in normal cells (P = 1.7E-03, Table 3). The second motif (maintenance motif, motif B in Figure 4) described triangles which pairs of hub-nodes (hub-n_1_, hub-n_2_) showed high correlation in one tissue type and no correlation in the other, while the mutual correlation of nodes n_1_-n_2 _stayed in the same category (no, low and high correlation). Such a scenario is reasonable for a mutated cancer protein with loss-of-function leaving their neighbors unaffected. Indeed, this motif occurred more often in the cancer networks (P = 6.34E-04, Table 3).

### Tumor networks are more robust against directed attacks

Albert and co-workers showed that scale free networks are error tolerant only against attacks of randomly selected nodes but not against directed removals of central nodes (hubs) [17]. We were interested in the robustness of the networks when removing their hubs. For this, we removed the most frequently involved nodes of every network and calculated the average of pair-wise distances (average network diameter) as an estimate of the fragility of the networks [17]. The relative increase of the network diameter due to the removal was distinctively larger in normal cells compared to cancer cells (average for cancer: 1.59, average for normal: 1.64, P = 0.021, Table 2) indicating higher robustness of the tumor networks against directed attacks at their hubs.

### Lower information content in normal cells

We used the number of pathways each single link was involved in (link-frequency) as an estimate of the probability that information (such as a phosphorylation) was passed through this link. In this simplified model, every pathway was treated equally. With this, we calculated the information content for each network. As a measure of disorder, Shannon's information entropy [18] was calculated for each network. The cancer networks exhibited a higher information entropy (average for cancer: 11.98, average for normal: 11.38, P = 3.28-04, Table 3) indicating their higher degree of dispersal.

### A comparative network motif

Inspired by the results described above, we designed a comparative network-motif which is illustrated in Figure 1. We wanted to put up a model in which cancer cells use different pathways for different tasks whereas normal cells use common signaling interactions for different tasks. Therefore a model was designed such that two pathways (two operator-receiver pairs, R_1 _- TF and R_2 _- TF in Figure 1) of the normal tissue shared at least one common link, whereas the same operator-receiver pairs for the tumor did not share any link. We compared the abundance of this motif with the abundance of its counterpart in which the *cancer *cells used at least one common link and the normal cells did not share any link. We found a significantly higher number of our motif in which the normal cells share a common link (average counts for cancer: 15,333,384, average for normal: 29,618,238, P = 9.54E-06, Table 3).

## Discussion

We investigated network properties of cancer signaling by looking at co-regulation patterns of genes for different cancer types. We analyzed the general regulatory behavior of correlating gene expression samples of one tumor type and study, rather than analyzing the regulatory behavior of single patients. For this, we calculated a gene to gene distance metric for all samples (patients) of normal and cancerous tissues. The networks of the investigated tumors showed distinctive mechanisms in the regulation of signal transduction when compared to normal cells and had shorter path lengths. Luscombe and co-workers analyzed the dynamics of regulatory networks in yeast [11]. In comparison to endogenously caused changes, they discovered a different topological adaptation of the network when yeast responded to environmental changes. For having quick responses, yeast reacted to environmental changes (nutrition depletion, stress response) by short regulatory cascades. Our investigated cancer cells showed a similar tendency as yeast under stress at which fine tuned endogenous homeostasis is of minor importance. Interestingly, for yeast, Luscombe *et al*. discovered a higher frequency of hubs for stress responses whereas we discovered that the tumors used hubs less frequently. Cells of normal sample had a more centralized network to regulate signals via common nodes and links. This was reflected by a smaller network, higher frequency of hubs, lower entropy and a higher number of our signaling motif in which the number of pathway-pairs with common links was counted. This makes sense, as fine-tuning and integrating diverse signals need to be coordinately transferred to the respective transcriptional response which is substantial for fine grained signaling homeostasis of normal cells to co-ordinate their signals in accordance to their cellular community in the tissue. Degenerated tumor cells do not need this any more. In turn, the tumors showed a higher connectedness of the whole network which may strengthen their independency of exogenic perturbations.

Similar to Cui and co-workers [12], we observed with our model that cancer specific mutations occur distinctively more often at hubs for signal transduction. Such a mutation can cause a loss of function. This is beneficial for the cancer if the protein gets insensitive to upstream-signals and fires constitutively an oncogenic signal as e.g. the ABL-BCR fusion protein in chronic myelogenous leukemia [19]. If the protein acts as a tumor suppressor, a complete loss of function is beneficial for oncogenesis. In both scenarios, the regulation for signaling homeostasis of the local network environment is detached from this mal-functional protein and a coordinated regulation between the environment and this protein is not necessary any more. We observed this by counting distinctively less integration-motifs in tumors (motif A in Figure 4). Interestingly, tumors seem to sustain the original signals between the environment. We observed this by higher counts of the disruption motif in tumors which reflects the disruption of co-regulation of the hub, but maintained regulation between the neighbors of the hubs (motif B in Figure 4). Even though tumors may exhibit de-regulation of mal-functional hubs with their neighbors, such a maintained co-regulation of their neighbors gives evidence that bypass regulations are still necessary. Ma'ayan and co-workers observed an accumulation of feedback and feed-forward loops at such hubs [20] which supports this idea. Tumors need to maintain the direct signal of e.g. a feed-forward loop which is necessary for the effect of the constitutive signal of an oncogenic hub (Figure 4C). Such oncogenic signaling motifs may have implications to drug therapy. If an oncogenic hub is treated (as e.g. ABL-BCR with imatinib [19]) resistance can occur by mutations of the target protein which reduce the affinity of the drug to the target. A combined therapy may avoid this evolvement by additionally blocking the signaling-maintenance of the neighbors. In addition, we found that the observed cancer networks showed higher error tolerance against directed attacks of hub removals. Hence, some maintenance signals may not only support cancer mutated hubs but also pave the way for the signaling network to get independent of them, specifically for proteins of cancer mutated genes with loss-of-function. It is challenging but highly relevant to shed light into these effects experimentally with cell lines exhibiting drug resistances at such hubs. We analyzed networks based on cohorts of patients and used the correlation of expression between gene pairs for the whole cohorts. This approach does not allow the analysis of a single sample and therefore can't be employed for diagnosis of a single patient, but rather for the analysis of tumor subgroups. It may be worthwhile developing distance metrics of gene pairs for single samples with which the investigated topology features can be employed supporting diagnosis.

We proposed a novel comparative signaling-motif for malignant signaling-regulation which sums up our findings (Figure 1). There have been elaborated studies on network motifs [21]. Our comparative cancer motif is different from these motifs in that it shows signaling-regulation in cancer reflecting less centralized formation. The comparative cancer motif agrees with our findings of non-integration (motif A, Figure 4) but signaling-maintenance (motif B, Figure 4) of proteins with higher involvement in signal propagation.

## Conclusion

We analyzed network models that based on correlation of gene expression between interacting proteins which enabled us to track basic principles of signaling by its regulation. The malignant signaling networks showed more diverse signaling pathways (average number of nodes in the networks of tumor: 3324, and normal tissue: 2973, P = 2.3E-03, Figure 2), shorter pathways (average path-length for cancer: 4.58, and normal: 5.50, P = 3.82E-05, Figure 2), the networks were less centralized (average clustering-coefficient of cancer: 0.118, and normal tissue: 0.125, P = 4.20E-04) and less dependent on hubs (average increase of network-diameter after hub-removal, for cancer: 1.59, and normal tissue: 1.64, P = 0.021). The cancer networks indicated signaling maintenance and increased error tolerance to punctual attacks even at hubs which makes cancer treatment at specific targets challenging.

## Methods

**Figure 5:**
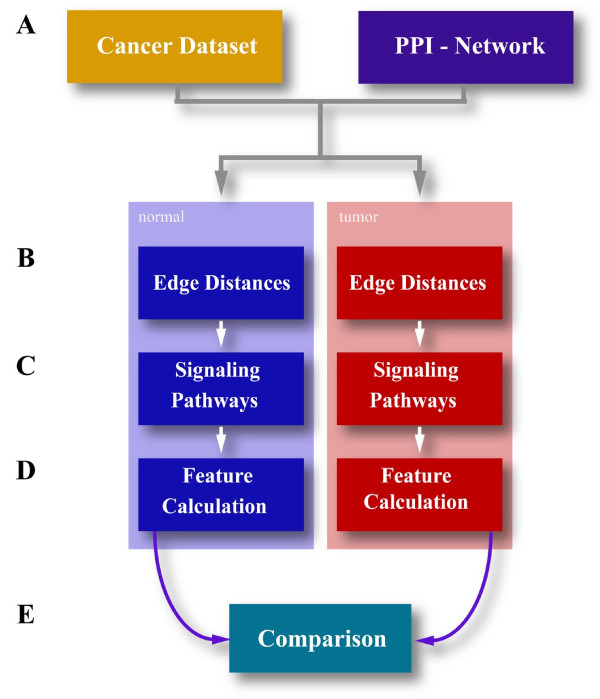
**Workflow of the method**. (A) Gene expression data from normal and tumor samples was mapped onto the respective nodes of the protein-protein-interaction network. (B) Node distances *d_xy _*were calculated from correlation coefficients of neighboring genes in the network for normal and tumor samples resulting in one normal and one tumor network with weighted links. Transcription factors and receptors were selected from public data repositories (Gene Ontology and TRANSFAC). (C) Shortest paths were calculated for all pair-wise combinations of receptors and transcription factors. Links and nodes that did not appear in any shortest path were removed and the largest connected component of the remaining network was used as the representative signaling network. (D) Network features were calculated for each signaling network and (E) the results for the networks of tumor and normal samples compared.

The general workflow of our approach is outlined in Figure 5. To investigate if our network features showed a statistically significant difference we performed paired Wilcoxon tests. We set the significance level to P ≤ 0.05 and considered all p-values below this threshold as statistically significant.

### Gene expression analysis

We analyzed twenty different datasets of cancer and their corresponding normal or reference samples. For most of the tumors (8 tumors), we analyzed two datasets for each cancer type. We used two AML (acute myeloid leukemia) datasets containing 18 normal and 25 tumor (AML-1) [22] and 4 normal and 52 cancer samples (AML-2) [23]. The first breast cancer dataset (breast-1) was obtained from cancer and normal sample of 43 patients each [24], breast-2 consisted of 143 normal and 42 cancer samples [25]. We analyzed two cervical cancer sets, cervical-1 [26] and cervical-2 [27] comprising data from 8 and 24 normal and 20 and 31 cancer datasets, respectively. Data of esophageal squamous cell carcinomas (ESCC) was obtained from cancerous and normal tissue of 53 patients (taken from the NCBI database Gene Expression Omnibus, accession code GSE23400). We used a glioma data set containing 23 normal and 153 cancer samples [28]. A head-and-neck dataset was taken from a study of head-and-neck squamous carcinoma consisting of data from 22 normal and cancer samples [29]. We used two lung cancer datasets, denoted as "lung-1" and "lung-2". Lung-1 was taken from a study by Bhattacharjee and co-workers [30] and contained data from 17 normal and 13 cancer samples of adenocarcinoma. Bhattacharjee and co-workers clustered the tumor datasets in their study. To obtain the most relevant data subsets with the necessary homogeneity, we selected their cluster of highly aggressive adenocarcinomas (cluster C2 of their cluster analysis) for our study. Lung-2 contained gene expression data of normal sample and adenocarcinoma tumors from 27 patients [31]. We analyzed an oral-tongue-cancer datasets comprising of data from 26 normal and 31 cancer samples (oral-tongue-1 [32]) and 12 and 26 normal and cancer samples, respectively (oral-tongue-2 [33]). We analyzed two datasets for pancreas cancer, pancreas-1 consisting of 39 normal and tumor tissues [34] and pancreas-2 having 15 normal and 36 cancer samples [35]. The first prostate cancer dataset (prostate-1) comprised of data from 50 normal sample and 52 cancer samples [36], and the second (prostate-2) consisted of 50 normal and 52 cancer samples (taken from the NCBI database Gene Expression Omnibus, accession code GSE17951). The dataset Renal-1 contained 23 normal renal samples and 69 samples of renal cancer 69 [37] and renal-2 had 5 normal and 62 cancer samples [38]. For the first renal datasets we selected homogenous samples by performing hierarchical clustering (Euclidean distance, complete linkage) yielding sets of nine clustered samples for normal tissue and 10 for cancerous tissue. We analyzed data from vulva interstitial neoplasia consisting of 10 normal and 9 cancer samples [39]. All datasets were stratified by randomly deleting datasets of the overrepresented class yielding an equal amount of tumor and normal sample datasets. For breast-1, ESCC, head-and-neck, lung-2, pancreas-1, and oral-tongue-1, normal and cancer samples were from the same patients (which was not the case for the other analyzed datasets). The data had been obtained using microarrays from Affymetrix of the following versions: HG-U133A for AML-1, breast-1, cervical-2, ESCC, lung-2 and renal-1, HG-U133 Plus 2 for breast-2, cervical-1, glioma, oral-tongue-2, pancreas-1, pancreas-2, prostate-2, renal-2 and vulva; HG-U95Av2 for AML-2, head-and-neck, lung-1, oral-tongue-1 and prostate-1. We normalized all datasets by Variance Stabilization Normalization [40,41].

### Network construction

The protein-protein-interaction network was constructed using the Human Protein Reference Database [13,14] (version 9 from April 13^th^, 2010). Interacting proteins were represented by their coding genes. The network was constructed for every gene that could be mapped to a microarray probe-set using BioMart [42]. Interactions were not taken into account if probe information for at least one gene was missing. For a link between node (gene) *x *and *y*, we defined a link-distance *d_xy _*by Pearson's correlation coefficient *ρ_xy _*from gene expression values of the interacting proteins *x *and *y*

(1) $$\rho_{xy}=\frac{\sum_{i=1}^{n}\left(x_{i}-\bar{x}\right)\left(y_{i}-\bar{y}\right)}{\sqrt{\sum_{i=1}^{n}{\left(x_{i}-\bar{x}\right)}^{2}\sum_{i=1}^{n}{\left(y_{i}-\bar{y}\right)}^{2}}}$$

(2) $$d_{xy}=1-\left|\rho_{xy}\right|$$

for *n *samples (patients) and gene expression *x_i _*and *y_i _*for gene *x *and *y *of sample *i*, respectively. These distances were calculated for each dataset of normal and cancer tissues and used for the networks of the respective datasets. To equally handle induction and inhibition events, we used the absolute values of all correlation coefficients. Correlation values were subtracted from one to obtain low distances for paths with high correlation. Genes with the molecular function term "receptor activity" from the definitions of Gene Ontology [43] were used as receptors in the network. The definitions of transcription factors were taken from TRANSFAC [44]. We used Dijkstra's algorithm [45] for calculating the shortest paths for every pair of receptors and transcription factors in the normal and tumor networks. These shortest paths of all receptor-transcription factor pairs served as the predicted pathways for each dataset and defined our tumor-specific interaction networks. Links and nodes that were not used by any shortest path were removed. The analyses were then performed on the largest connected component of the interaction network.

### Defining the network features

Path length, link and node frequency, and the signaling motif are explained in the results. It is to note that link (and node) frequency is similar to betweenness centrality, which is the number of shortest paths going through the link (and node). While betweenness centrality considers shortest paths between all pairs of nodes, node and link frequency as defined here, was the number of shortest paths between pairs of receptors and transcription factors. The (average) network diameter has been described as a measure for error tolerance of a network against removals of nodes in scale free networks [17] and was used here in a similar way. The diameters for the networks were obtained by the average of the shortest paths of each pair of nodes in the network. The network diameter was calculated for undisturbed (whole) networks and networks in which the top 10% of the hubs were removed. The ratio of these values was calculated to yield the increase of the average network diameter after hub removal. The calculation of the information content was based on the assumption that signals enter the network at any receptor with equal probability within a certain time interval. These signals are passed by the links of the network to the transcription factors via the defined pathways from the receptors, again with equal probability. We assumed that the signals vanish from the signaling network after having entered the corresponding transcription factor at the end of the path. Signals enter the receptors with a certain frequency, resulting in an equal distribution and therefore we assumed uniform density of the signals in each pathway. The probability of a signal to pass through the link of node *i *and *j *is then proportional to the number of pathways passing through this link. With this, we calculated the information content by Shannon's definition [18]

(3) $$I=-\sum_{i=1}^{n}p_{i}\log_{2}\left(p_{i}\right)$$

in which *n *denotes the number of links and *p_i _*the probability of a signal to be passed through link *i*. The clustering coefficient *C_i _*for node *i *was given by

(4) $$C_{i}=\frac{n_{links}}{k\left(k-1\right)}$$

in which *n_links _*is the number of links connecting the neighbors of node *i *and *k *is the number of neighbors. This feature described how well the neighbors were mutually connected. If they were fully connected, the clustering coefficient was one, if they were not connected at all, the clustering coefficient was zero.

### Link-frequency distributions

The link-frequency distributions of normal and tumor cells *i *followed a power law, i.e. the probability of links *P(f) *with link-frequency *f *was approximately given by

(5) $$P\left(f\right)\sim f^{-\propto }$$

To estimate the exponent *α *we applied the method proposed by Newman [46] which determines the exponent of the cumulative distribution avoiding noisy data at the tail of the original distribution (see tail of the link frequency distribution in Figure 3). For visualization we plotted the distribution and the corresponding linear function with slope *α *on a log-log scale. The intersection with the y-axis of the plotted line was calculated using a least squared fit (see Figure 3 and Additional file 1: Supplemental Figure S2).

### Defining and counting the integration and the maintenance motif

We defined three correlation categories based on intervals of the absolute values of the correlation coefficient |*ρ_xy_*|: no correlation for the absolute value of correlation coefficients between zero and 0.3, low correlation for the absolute value of correlation coefficients between 0.3 and 0.5, and high correlation above 0.5. Hubs of cancer mutated genes were defined by intersecting the list of cancer genes from Cui and co-workers (Supplementary Table S10 in [12]) with the nodes that appeared in both tissue types (normal and tumor). From this intersection we selected the top 50 most frequently involved nodes from the normal and the tumor network resulting in 100 cancer mutated hubs for every cancer dataset. Hubs that were selected in both tissue types and as such appeared twice in the union set were used only once. For each dataset, we collected all triangles in which one node was such a cancer mutated hub and that appeared in the normal and in the tumor network ensuring the comparability of our motif counts. Out of these triangles, we selected triangles having the motifs for integration (motif A in Figure 4) and maintenance (motif B in Figure 4). For motif A, we selected triangles in which the absolute correlations |*ρ_xy_*| between all pairs of nodes (hub-n_1_, hub-n_2_, n_1_-n_2_, n_1 _and n_2 _are the two other nodes in the triangle) was high. For motif B, we counted the abundance of triangles which pairs of hub-nodes showed high correlation in one tissue type and no correlation in the other, while the correlation of n_1_-n_2 _stayed in the same category (no correlation, low correlation or high correlation).

## Authors' contributions

GS, NK and RK conceived the study and drafted the manuscript. RK guided the study and proof-read the manuscript. All authors read and approved the final manuscript.

## Acknowledgements

We thank Tim Beissbarth for his suggestions for the statistical analysis, and Tobias Bauer for technical support. This work was funded by the Helmholtz Alliance on Systems Biology of Signaling in Cancer, the *Nationales Genom-Forschungs-Netz *(NGFN+) for the project ENGINE and the Helmholtz International Graduate School for Cancer Research at the German Cancer Research Center.

## Supplementary Material

Additional file 1 **Additional results**. Results of the enrichment analysis of networks and network hubs, intersection of hubs and cancer mutated genes, distribution of correlation coefficients and link frequency for normal and tumor samples for all cancer data sets, and visualizations of smaller sub networks. Click here for file
