# Supplementary material for: Regulation patterns in signaling networks of cancer
Source: BMC Syst Biol. 2010 Nov 26;4:162. doi: 10.1186/1752-0509-4-162 (PMC3225866; doi:10.1186/1752-0509-4-162)
Supplement: Additional file 1 — Additional results. Results of the enrichment analysis of networks and network hubs, intersection of hubs and cancer mutated genes, distribution of correlation coefficients and link frequency for normal and tumor samples for all cancer data sets, and visualizations of smaller sub networks. [file 1752-0509-4-162-S1.PDF]

Additional file 1

Supplemental Table S1. Tumor specific networks are enriched with somatically mutated genes (taken from the database COSMIC)

	COSMIC genes in tumor network	COSMIC genes not in tumor network	Tumor network genes not in COSMIC	Genes not in tumor network and not in COSMIC	P-value (Fisher's exact test)
**AML 1**	60	10	3467	3673	1.10E-10
**AML 2**	60	5	2869	2610	3.95E-12
**Breast 1**	3198	3600	142	270	3.38E-07
**Breast 2**	3306	4740	155	414	1.87E-11
**Cervical 1**	49	8	3702	4856	3.42E-11
**Cervical 2**	47	9	3336	3818	9.62E-09
**ESCC**	52	8	3553	3597	2.28E-09
**Glioma**	19	3	3609	4984	2.39E-05
**Head and neck**	1206	1031	1337	1970	3.14E-23
**Lung 1**	1284	965	1407	1888	3.71E-26
**Lung 2**	1507	1241	1790	2672	2.37E-34
**Oral-tongue 1**	1331	918	1478	1817	5.86E-26
**Oral-tongue 2**	255	138	3452	4770	3.70E-19
**Pancreas 1**	1573	1457	2114	3471	1.97E-36
**Pancreas 2**	1596	1434	2155	3430	1.94E-36
**Prostate 1**	40	18	2270	3216	2.23E-05
**Prostate 2**	51	11	3709	4844	4.18E-10
**Renal 1**	1453	1289	1756	2712	5.00E-30
**Renal 2**	1574	1525	1984	3532	5.60E-41
**Vulva**	48	7	3339	5221	1.98E-13

Supplemental Table S2. Cancer mutated genes are significantly enriched in the 50 most frequently involved nodes (hubs)

	Number of nodes		Cancer genes in the whole network		Cancer genes in first 50 hubs		P-value	
	normal	tumor	normal	tumor	normal	tumor	normal	tumor
**AML 1**	2831	3536	255	282	20	19	2.40E-09	8.99E-08
**AML 2**	2028	2938	238	294	17	16	2.39E-05	1.67E-03
**Breast 1**	3310	3348	299	294	16	18	3.80E-06	9.31E-08
**Breast 2**	3384	3470	271	275	18	13	1.83E-08	1.16E-04
**Cervical 1**	2904	3761	249	294	13	17	1.96E-04	3.46E-06
**Cervical 2**	3129	3389	284	288	10	13	1.25E-02	4.03E-04
**ESCC**	3303	3613	279	312	12	18	6.78E-04	2.41E-07
**Glioma**	3386	3635	281	288	15	13	6.60E-06	1.85E-04
**Head and neck**	2555	2550	270	261	13	13	1.48E-03	1.08E-03
**Lung 1**	2713	2698	274	278	19	18	1.11E-07	7.85E-07
**Lung 2**	3215	3305	263	291	14	18	2.72E-05	1.24E-07
**Oral tongue 1**	2594	2816	264	286	15	18	7.39E-05	2.32E-06
**Oral tongue 2**	3485	3715	278	295	17	13	1.17E-07	1.77E-04
**Pancreas 1**	2903	3694	238	299	17	14	1.64E-07	3.36E-04
**Pancreas 2**	2868	3758	248	290	15	11	1.06E-05	9.51E-03
**Prostate 1**	2203	2316	244	245	7	8	0.31	0.18
**Prostate 2**	3548	3769	293	313	12	11	5.56E-04	3.42E-03
**Renal 1**	3196	3216	280	284	18	18	7.41E-08	9.26E-08
**Renal 2**	2548	3566	231	281	12	13	1.24E-03	2.20E-03
**Vulva**	3356	3394	289	278	15	18	1.04E-05	3.12E-08

Supplemental Table S3 Intersection of our hubs and cancer mutated genes

	Gene symbols (RefSeq IDs in brackets) of the intersection of the top 50 most frequently involved genes (hubs) and cancer mutated genes from table S10 of Cui and co-workers (Mol Sys Biol, 2007, 3:152)
**AML-1 normal**	LCK (NP_005347) CREBBP (NP_004371) FYN (NP_002028) PRKAR1A (NP_997636) TNF (NP_000585) EP300 (NP_001420) XDH (NP_000370) HOXA9 (NP_689952) TGFBR2 (NP_003233) HSP90AA1 (NP_001017963) AC046176.2 (NP_002341) PRKCA (NP_002728) CDK2 (NP_001789) XRCC6 (NP_001460) RARA (NP_001019980) MYC (NP_002458) JAK2 (NP_004963) TGFBR1 (NP_001124388) EGFR (NP_005219) JAK1 (NP_002218)
**AML-1 tumor**	CREBBP (NP_004371) EGFR (NP_005219) SMAD2 (NP_005892) TNF (NP_000585) SMAD4 (NP_005350) EP300 (NP_001420) CTNNB1 (NP_001895) SPTAN1 (NP_001123910) FYN (NP_002028) PRKAR1A (NP_997636) LCK (NP_005347) STK11 (NP_000446) BRCA1 (NP_009228) SMAD3 (NP_005893) RARA (NP_001019980) MAPK7 (NP_620602) JAK1 (NP_002218) PTK6 (NP_005966) XRCC6 (NP_001460)
**AML-2 normal**	SPTAN1 (NP_001123910) BRCA1 (NP_009228) EP300 (NP_001420) CREBBP (NP_004371) CTNNB1 (NP_001895) FYN (NP_002028) SMAD2 (NP_005892) ROCK2 (NP_004841) MKL1 (NP_065882) SMAD4 (NP_005350) PKN2 (NP_006247) CSK (NP_004374) LCK (NP_005347) TNF (NP_000585) DAXX (NP_001135442) TNK2 (NP_001010938) FOXO1 (NP_002006)
**AML-2 tumor**	EP300 (NP_001420) TNF (NP_000585) ABL1 (NP_009297) CREBBP (NP_004371) CTNNB1 (NP_001895) PRKAR1A (NP_997636) SMAD2 (NP_005892) SMAD3 (NP_005893) RARA (NP_001019980) SPTAN1 (NP_001123910) HSP90AA1 (NP_001017963) STK11 (NP_000446) LCK (NP_005347) FYN (NP_002028) BTK (NP_000052) AC023165.1 (NP_444259)
**Breast-1 normal**	SMAD2 (NP_005892) RARA (NP_001019980) EP300 (NP_001420) TNF (NP_000585) SMAD3 (NP_005893) EGFR (NP_005219) SPTAN1 (NP_001123910) CREBBP (NP_004371) PRKAR1A (NP_997636) CTNNB1 (NP_001895) BTK (NP_000052) PRKACB (NP_997461) MAPK7 (NP_620602) HNF1A (NP_000536) PTK6 (NP_005966) SMAD4 (NP_005350)
**Breast-1 tumor**	SPTAN1 (NP_001123910) PRKAR1A (NP_997636) EGFR (NP_005219) EP300 (NP_001420) SMAD3 (NP_005893) CREBBP (NP_004371) TNF (NP_000585) RARA (NP_001019980) SMAD2 (NP_005892) BTK (NP_000052) PTK6 (NP_005966) CTNNB1 (NP_001895) MAPK7 (NP_620602) SMAD4 (NP_005350) RUNX1 (NP_001116079) MAPK14 (NP_001306) STK11 (NP_000446) HNF1A (NP_000536)
**Breast-2 normal**	TNF (NP_000585) SMAD4 (NP_005350) CREBBP (NP_004371) SMAD3 (NP_005893) EP300 (NP_001420) MKL1 (NP_065882) PRKAR1A (NP_997636) BTK (NP_000052) SMAD2 (NP_005892) FYN (NP_002028) STK11 (NP_000446) PTK6 (NP_005966) HSP90AA1 (NP_001017963) JAK1 (NP_002218) PRKCZ (NP_001028754) SMARCA4 (NP_003063) CTNNB1 (NP_001895) XRCC6 (NP_001460)
**Breast-2 tumor**	CREBBP (NP_004371) TNF (NP_000585) MAPK14 (NP_001306) SPTAN1 (NP_001123910) LCK (NP_005347) FYN (NP_002028) MKL1 (NP_065882) EP300 (NP_001420) STK11 (NP_000446) PTK6 (NP_005966) PRKAR1A (NP_997636) XRCC6 (NP_001460) SMAD3 (NP_005893)
**Cervical-1 normal**	MKL1 (NP_065882) EGFR (NP_005219) CREBBP (NP_004371) SPTAN1 (NP_001123910) MAPK7 (NP_620602) HSP90AA1 (NP_001017963) TSC2 (NP_001070651) SMAD2 (NP_005892) MET (NP_001120972) SMAD4 (NP_005350) XRCC6 (NP_001460) HOXA9 (NP_689952) ROCK1 (NP_005397)
**Cervical-1 tumor**	SPTAN1 (NP_001123910) CREBBP (NP_004371) EP300 (NP_001420) LCK (NP_005347) FYN (NP_002028) TNF (NP_000585) SMAD4 (NP_005350) ADRBK1 (NP_001610) SMAD2 (NP_005892) JAK2 (NP_004963) XRCC6 (NP_001460) BIRC6 (NP_057336) EGFR (NP_005219) BRCA1 (NP_009228) CTNNB1 (NP_001895) HSP90AA1 (NP_001017963) PRKAR1A (NP_997636)
**Cervical-2 normal**	CTNNB1 (NP_001895) BIRC6 (NP_057336) CREBBP (NP_004371) SMAD2 (NP_005892) FYN (NP_002028) EP300 (NP_001420) SMAD4 (NP_005350) TGFBR1 (NP_001124388) NCOA2 (NP_006531) JAK1 (NP_002218)
**Cervical-2 tumor**	SMAD2 (NP_005892) TNF (NP_000585) SMAD3 (NP_005893) CREBBP (NP_004371) CTNNB1 (NP_001895) PRKAR1A (NP_997636) TIAM1 (NP_003244) LCK (NP_005347) AC046176.2 (NP_002341) XRCC6 (NP_001460) PRKCB (NP_997700) HSP90AA1 (NP_001017963) EP300 (NP_001420)
**ESCC normal**	TNF (NP_000585) SMAD3 (NP_005893) FYN (NP_002028) PRKAR1A (NP_997636) TGFBR1 (NP_001124388) EP300 (NP_001420) SMAD4 (NP_005350) RARA (NP_001019980) BRCA1 (NP_009228) PTK6 (NP_005966) CREBBP (NP_004371) SMAD2 (NP_005892)
**ESCC tumor**	EP300 (NP_001420) CREBBP (NP_004371) TNF (NP_000585) PRKAR1A (NP_997636) RARA (NP_001019980) FYN (NP_002028) SMAD3 (NP_005893) CTNNB1 (NP_001895) SPTAN1 (NP_001123910) SMAD2 (NP_005892) LCK (NP_005347) SMAD4 (NP_005350) BRCA1 (NP_009228) JAK1 (NP_002218) EGFR (NP_005219) HSP90AA1 (NP_001017963) PTK6 (NP_005966) MAPK7 (NP_620602)
**Glioma normal**	EP300 (NP_001420) SMAD4 (NP_005350) SMAD3 (NP_005893) CREBBP (NP_004371) NF2 (NP_861968) BIRC6 (NP_057336) MKL1 (NP_065882) FYN (NP_002028) CTNNB1 (NP_001895) MAPK14 (NP_001306) PRKAR1A (NP_997636) RARA (NP_001019980) FOXO1 (NP_002006) BTK (NP_000052) STK11 (NP_000446)
**Glioma tumor**	TNF (NP_000585) CREBBP (NP_004371) PRKCA (NP_002728) STK11 (NP_000446) EP300 (NP_001420) AC046176.2 (NP_002341) RARA (NP_001019980) PKN2 (NP_006247) BTK (NP_000052) SMAD4 (NP_005350) SMAD2 (NP_005892) CTNNB1 (NP_001895) CDK2 (NP_001789)

**Head-and-neck normal**	SMAD2 (NP_005892) FYN (NP_002028) CTNNB1 (NP_001895) RARA (NP_001019980) BRCA1 (NP_009228) CREBBP (NP_004371) SMAD4 (NP_005350) RUNX1 (NP_001116079) EP300 (NP_001420) PER1 (NP_002607) AC023165.1 (NP_444259) RYK (NP_002949) HSP90AA1 (NP_001017963)
**Head-and-neck tumor**	CREBBP (NP_004371) FYN (NP_002028) CTNNB1 (NP_001895) SMAD4 (NP_005350) SMAD2 (NP_005892) RARA (NP_001019980) PER1 (NP_002607) RUNX1 (NP_001116079) LCK (NP_005347) EP300 (NP_001420) SMAD3 (NP_005893) PRKCB (NP_997700) BIRC6 (NP_057336)
**Lung-1 normal**	HSP90AA1 (NP_001017963) SPTAN1 (NP_001123910) TNF (NP_000585) CREBBP (NP_004371) EP300 (NP_001420) BTK (NP_000052) RARA (NP_001019980) CTNNB1 (NP_001895) SMAD4 (NP_005350) PRKCZ (NP_001028754) AC046176.2 (NP_002341) MAPK14 (NP_001306) SMAD3 (NP_005893) SMAD2 (NP_005892) CHUK (NP_001269) PRKAR1A (NP_997636) ERBB2 (NP_004439) MYC (NP_002458) FYN (NP_002028)
**Lung-1 tumor**	RARA (NP_001019980) CREBBP (NP_004371) JAK1 (NP_002218) EP300 (NP_001420) PRKCA (NP_002728) CTNNB1 (NP_001895) TNF (NP_000585) SMAD3 (NP_005893) SMAD2 (NP_005892) PTK6 (NP_005966) BRCA1 (NP_009228) BTK (NP_000052) SMAD4 (NP_005350) PRKAR1A (NP_997636) JAK2 (NP_004963) FYN (NP_002028) NCOA2 (NP_006531) HOXA9 (NP_689952)
**Lung-2 normal**	SMAD2 (NP_005892) AC023165.1 (NP_444259) FYN (NP_002028) HSP90AA1 (NP_001017963) MKL1 (NP_065882) BIRC6 (NP_057336) EP300 (NP_001420) CREBBP (NP_004371) LCK (NP_005347) BCL2 (NP_000648) JAK1 (NP_002218) PTK6 (NP_005966) SMAD4 (NP_005350) KDR (NP_002244)
**Lung-2 tumor**	CTNNB1 (NP_001895) SMAD2 (NP_005892) CAMK4 (NP_001735) MKL1 (NP_065882) CREBBP (NP_004371) MAPK7 (NP_620602) JAK1 (NP_002218) SMAD3 (NP_005893) XRCC6 (NP_001460) EP300 (NP_001420) BRCA1 (NP_009228) AC023165.1 (NP_444259) TNF (NP_000585) HIPK2 (NP_073577) FYN (NP_002028) LCK (NP_005347) PRKCZ (NP_001028754) AC046176.2 (NP_002341)
**Oral-Tongue-1 normal**	PRKAR1A (NP_997636) CTNNB1 (NP_001895) HSP90AA1 (NP_001017963) RB1 (NP_000312) CREBBP (NP_004371) BRCA1 (NP_009228) BTK (NP_000052) SMAD2 (NP_005892) SMAD4 (NP_005350) LCK (NP_005347) TNF (NP_000585) STK11 (NP_000446) SPTAN1 (NP_001123910) MAPK7 (NP_620602) RUNX1 (NP_001116079)
**Oral-Tongue-1 tumor**	CREBBP (NP_004371) LCK (NP_005347) EP300 (NP_001420) SMAD4 (NP_005350) TNF (NP_000585) TCF12 (NP_996919) SMAD2 (NP_005892) PRKCA (NP_002728) SMAD3 (NP_005893) PRKAR1A (NP_997636) FYN (NP_002028) SPTAN1 (NP_001123910) HSP90AA1 (NP_001017963) RARA (NP_001019980) CTNNB1 (NP_001895) ADRBK1 (NP_001610) ITK (NP_005537) BTK (NP_000052)
**Oral-Tongue-2 normal**	CREBBP (NP_004371) SMAD2 (NP_005892) MAPK8 (NP_620635) PRKAR1A (NP_997636) SMAD3 (NP_005893) EP300 (NP_001420) CTNNB1 (NP_001895) LCK (NP_005347) BIRC6 (NP_057336) FYN (NP_002028) TNF (NP_000585) KDR (NP_002244) MAPK7 (NP_620602) MKL1 (NP_065882) MYC (NP_002458) TGFBR1 (NP_001124388) FOXO1 (NP_002006)
**Oral-Tongue-2 tumor**	SMAD2 (NP_005892) RARA (NP_001019980) EP300 (NP_001420) CREBBP (NP_004371) SPTAN1 (NP_001123910) CTNNB1 (NP_001895) MKL1 (NP_065882) BTK (NP_000052) TNF (NP_000585) XRCC6 (NP_001460) CDK4 (NP_000066) STK11 (NP_000446) HSP90AA1 (NP_001017963)
**Pancreas-1 normal**	FYN (NP_002028) RARA (NP_001019980) SMAD2 (NP_005892) CASP8 (NP_001219) EP300 (NP_001420) AC046176.2 (NP_002341) PRKCZ (NP_001028754) CREBBP (NP_004371) CSNK1A1 (NP_001020276) JAK2 (NP_004963) TNF (NP_000585) MAPK14 (NP_001306) BTK (NP_000052) STK11 (NP_000446) CREB1 (NP_604391) LCK (NP_005347) CTNNB1 (NP_001895)
**Pancreas-1 tumor**	SMAD2 (NP_005892) CREBBP (NP_004371) EP300 (NP_001420) TNF (NP_000585) SPTAN1 (NP_001123910) AC046176.2 (NP_002341) FYN (NP_002028) SMAD4 (NP_005350) STK11 (NP_000446) TCF12 (NP_996919) SMAD3 (NP_005893) PRKCA (NP_002728) XRCC6 (NP_001460) CTNNB1 (NP_001895)
**Pancreas-2 normal**	SMAD2 (NP_005892) BTK (NP_000052) RARA (NP_001019980) FYN (NP_002028) TNF (NP_000585) JAK2 (NP_004963) EP300 (NP_001420) AC046176.2 (NP_002341) PRKACB (NP_997461) LCK (NP_005347) CREB1 (NP_604391) CSNK1A1 (NP_001020276) STK11 (NP_000446) CTNNB1 (NP_001895) CASP8 (NP_001219)
**Pancreas-2 tumor**	FYN (NP_002028) EP300 (NP_001420) SMAD3 (NP_005893) CREBBP (NP_004371) TNF (NP_000585) SMAD2 (NP_005892) SPTAN1 (NP_001123910) STK11 (NP_000446) RARA (NP_001019980) CTNNB1 (NP_001895) PRKAR1A (NP_997636)
**Prostate-1 normal**	HSP90AA1 (NP_001017963) CTNNB1 (NP_001895) BTK (NP_000052) TNF (NP_000585) RYK (NP_002949) CREBBP (NP_004371) SMARCA4 (NP_003063)
**Prostate-1 tumor**	RB1 (NP_000312) HSP90AA1 (NP_001017963) CTNNB1 (NP_001610) CREBBP (NP_004371) SMARCA4 (NP_003063) TNF (NP_000585) ADRBK1 (NP_001610) RYK (NP_002949)
**Prostate-2 normal**	CREBBP (NP_004371) TNF (NP_000585) EP300 (NP_001420) CTNNB1 (NP_001895) LCK (NP_005347) RARA (NP_001019980) SPTAN1 (NP_001123910) XRCC6 (NP_001460) SMAD2 (NP_005892) SMAD3 (NP_005893) XDH (NP_000370) JAK2 (NP_004963)
**Prostate-2 tumor**	SPTAN1 (NP_001123910) CREBBP (NP_004371) SMAD3 (NP_005893) TNF (NP_000585) EP300 (NP_001420) BRCA1 (NP_009228) FYN (NP_002028) PRKAR1A (NP_997636) SMAD2 (NP_005892) CTNNB1 (NP_001895) STK11 (NP_000446)
**Renal-1 normal**	SPTAN1 (NP_001123910) EP300 (NP_001420) SMAD3 (NP_005893) EGFR (NP_005219) TNF (NP_000585) CREBBP (NP_004371) XRCC6 (NP_001460) PTK6 (NP_005966) NCOA2 (NP_006531) PRKAR1A (NP_997636) MAPK14 (NP_001306) PTK2B (NP_004094) CTNNB1 (NP_001895) TSC1 (NP_001155898) BRCA1 (NP_009228) MKL1 (NP_065882) AC046176.2 (NP_002341) MYC (NP_002458)
**Renal-1 tumor**	TNF (NP_000585) EP300 (NP_001420) SMAD2 (NP_005892) JAK2 (NP_004963) SPTAN1 (NP_001123910) LCK (NP_005347) CREBBP (NP_004371) EGFR (NP_005219) MAPK14 (NP_001306) BRCA1 (NP_009228) PRKAR1A (NP_997636) CTNNB1 (NP_001895) PTK2B (NP_004094) JAK1 (NP_002218) PER1 (NP_002607) SMAD3 (NP_005893) SMAD4 (NP_005350) HIPK2 (NP_073577)
**Renal-2 normal**	TNF (NP_000585) FYN (NP_002028) SMAD2 (NP_005892) SMAD4 (NP_005350) PLCG1

	(NP_002651) PER1 (NP_002607) CDK4 (NP_000066) SPTAN1 (NP_001123910) CSK (NP_004374) RUNX1 (NP_001116079) CREBBP (NP_004371) MKL1 (NP_065882)
**Renal-2 tumor**	EP300 (NP_001420) CTNNB1 (NP_001895) LCK (NP_005347) SMAD2 (NP_005892) CREBBP (NP_004371) RARA (NP_001019980) SPTAN1 (NP_001123910) SMAD4 (NP_005350) TNF (NP_000585) BTK (NP_000052) PRKAR1A (NP_997636) FYN (NP_002028) CSNK1A1 (NP_001020276)
**Vulva normal**	RARA (NP_001019980) TNF (NP_000585) PRKAR1A (NP_997636) LCK (NP_005347) NF2 (NP_861968) XIAP (NP_001158) SMAD4 (NP_005350) CTNNB1 (NP_001895) CREBBP (NP_004371) TIAM1 (NP_003244) SPTAN1 (NP_001123910) BRCA1 (NP_009228) SMAD3 (NP_005893) FYN (NP_002028) RUNX1 (NP_001116079)
**Vulva tumor**	SMAD3 (NP_005893) LCK (NP_005347) CDK4 (NP_000066) FYN (NP_002028) PRKACB (NP_997461) CTNNB1 (NP_001895) EP300 (NP_001420) BTK (NP_000052) MKL1 (NP_065882) PRKCA (NP_002728) CREBBP (NP_004371) TNF (NP_000585) XDH (NP_000370) CHUK (NP_001269) PRKCZ (NP_001028754) BRCA1 (NP_009228) SPTAN1 (NP_001123910) RARA (NP_001019980)

Supplemental Figure S1

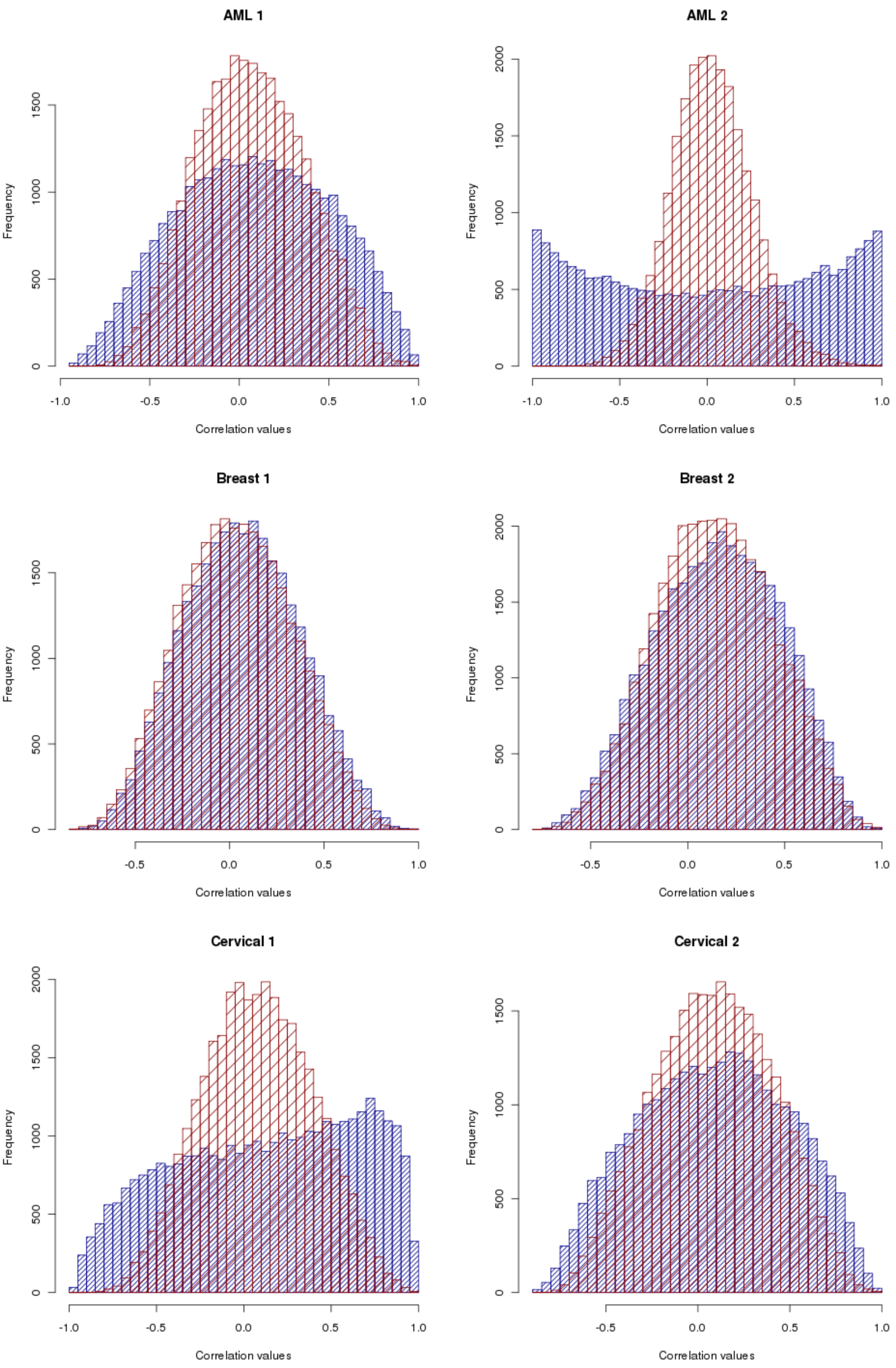

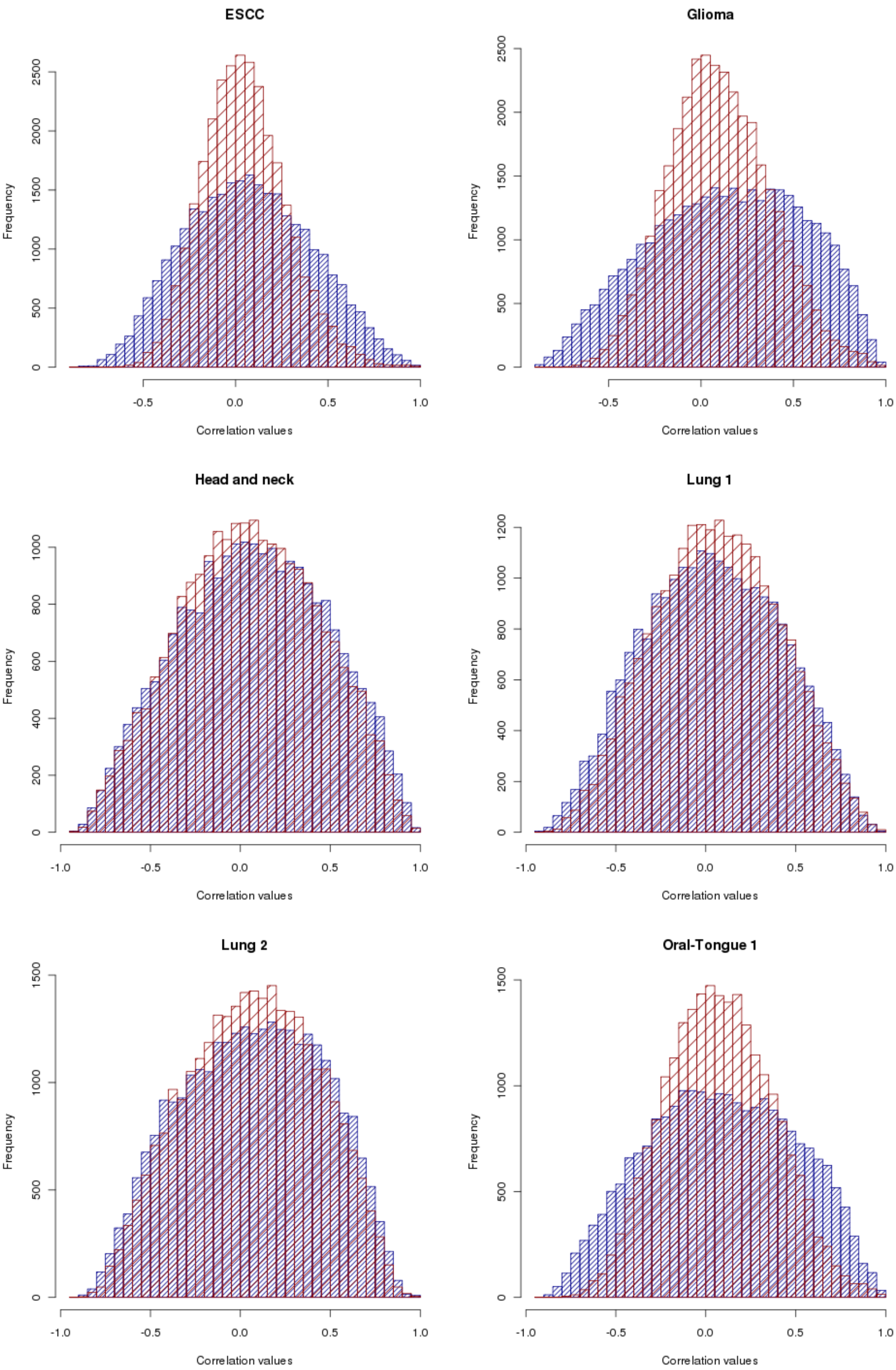
ESCC
Glioma
Head and neck
Lung 1
Lung 2
Oral-Tongue 1
Frequency
Correlation values

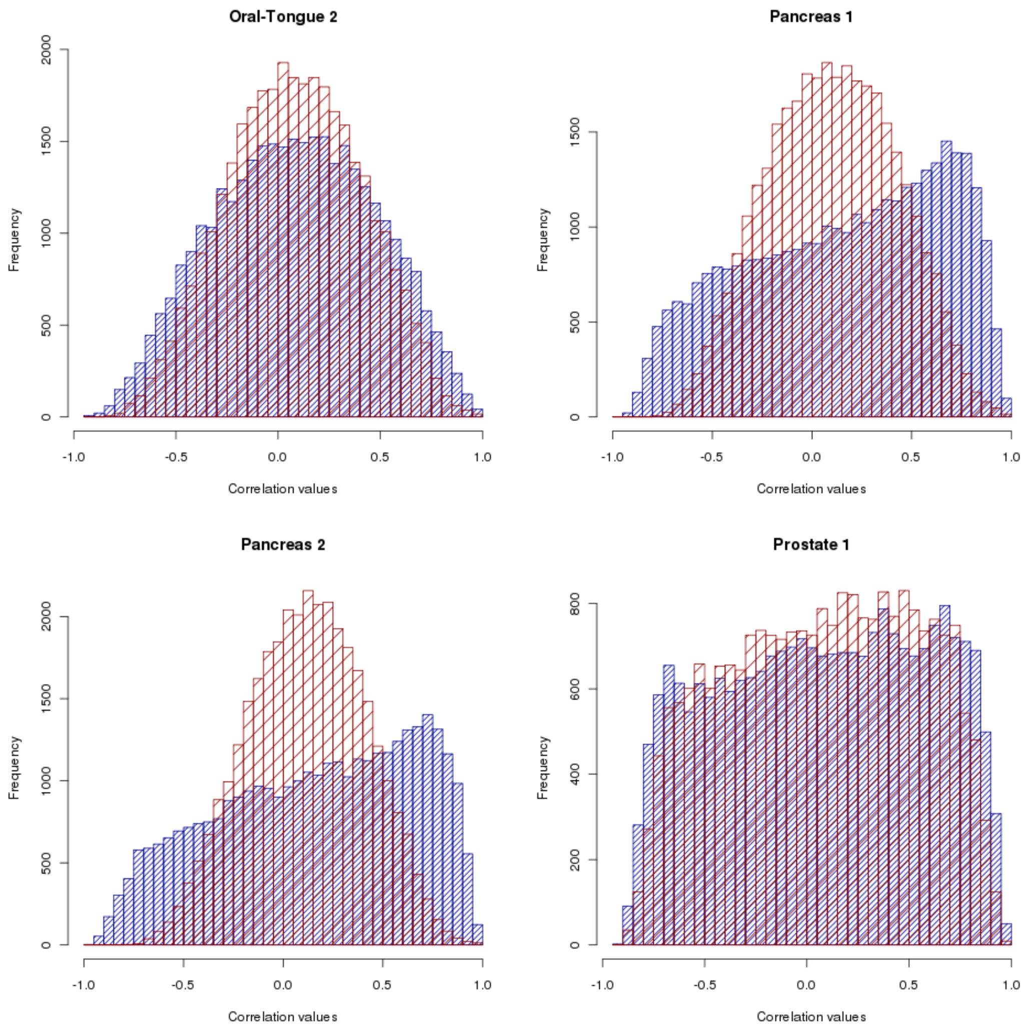
Oral-Tongue 2
Pancreas 1
Pancreas 2
Prostate 1
Frequency
Correlation values
-1.0
-0.5
0.0
0.5
1.0
0
500
1000
1500
2000
200
400
600
800

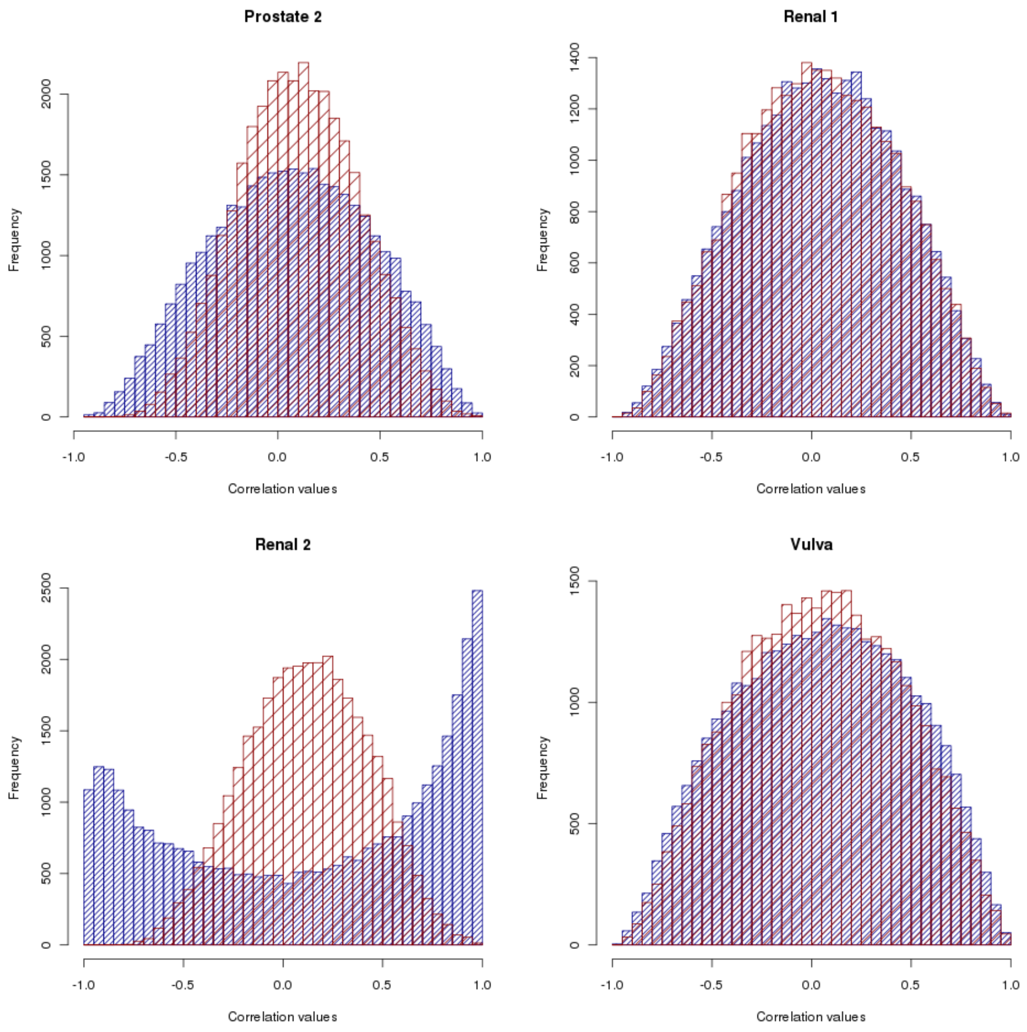

Figure S1. Distribution of the correlation coefficients for all analyzed datasets (blue bars: normal, red bars: cancer).

Supplemental Figure S2. Distributions of link-frequency for all datasets

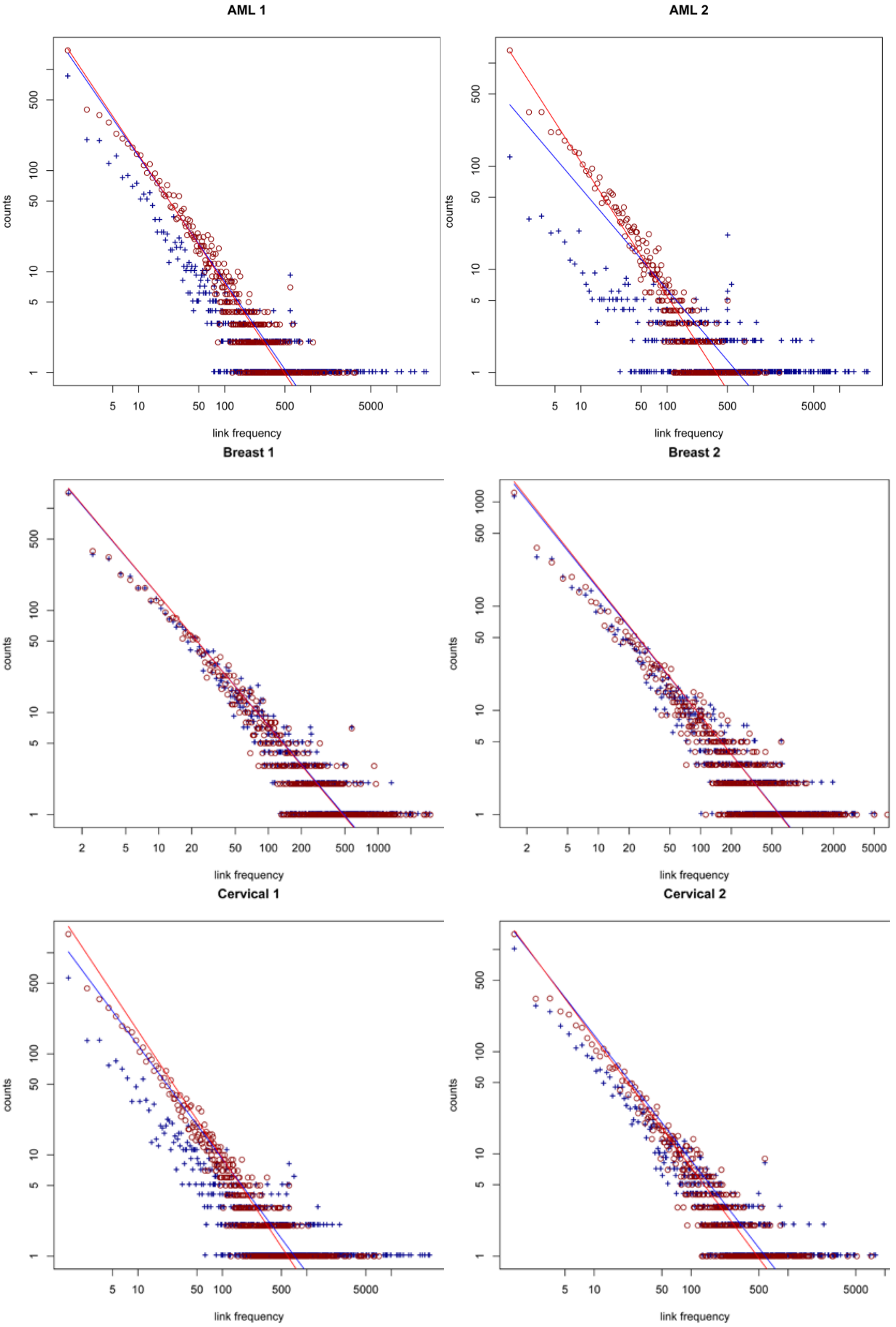

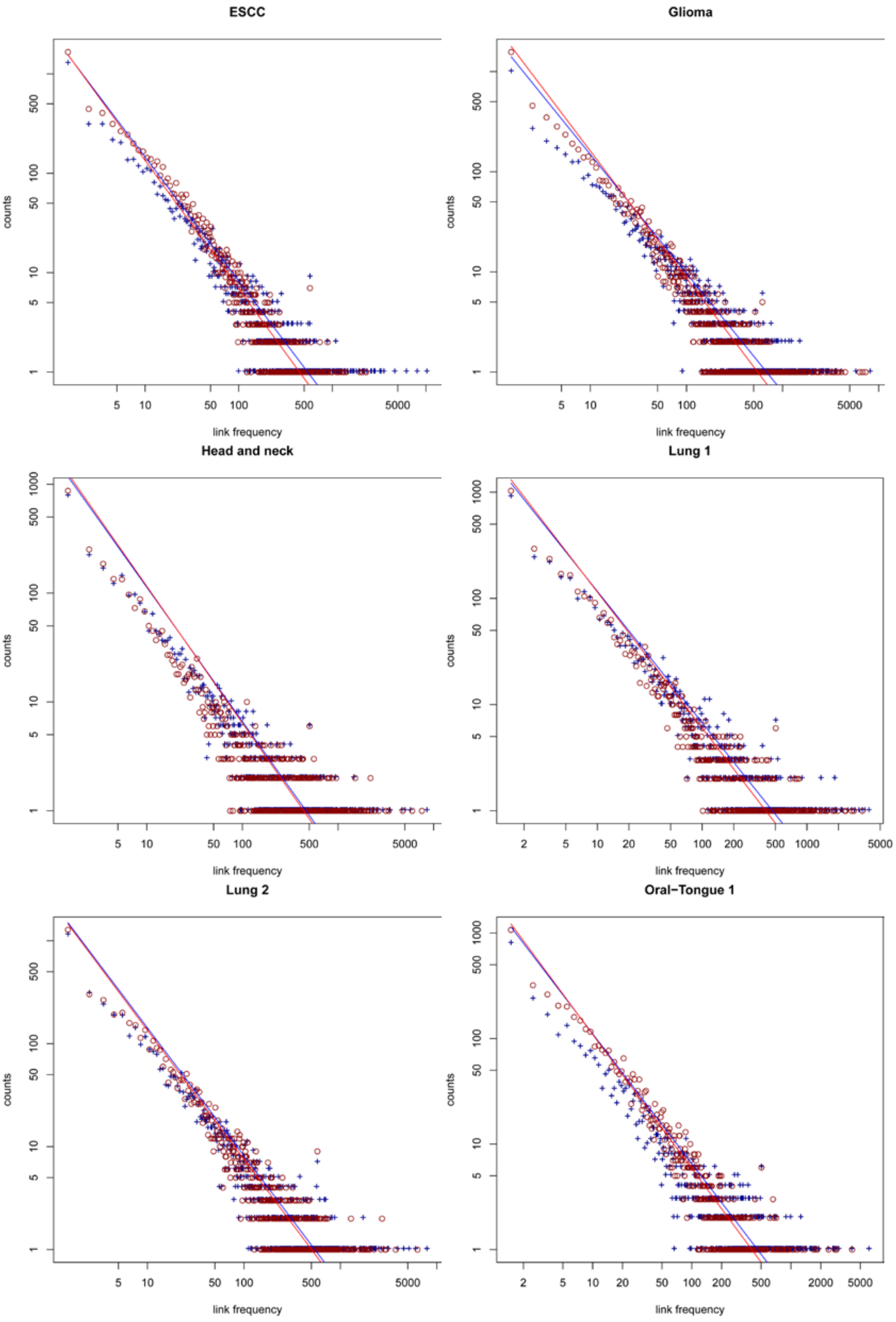
ESCC
Glioma
Head and neck
Lung 1
Lung 2
Oral−Tongue 1
counts
link frequency

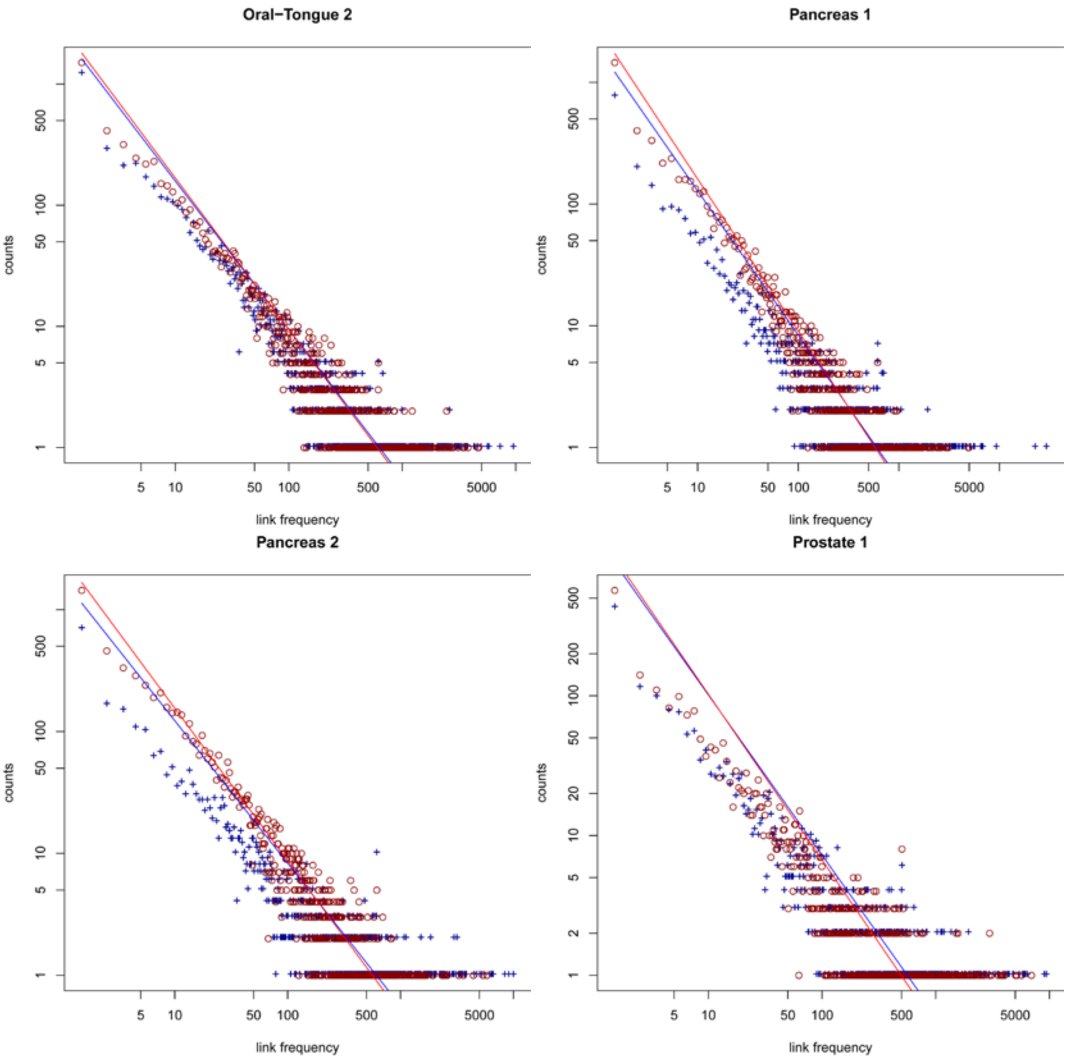
Oral−Tongue 2
Pancreas 1
Pancreas 2
Prostate 1
counts
link frequency

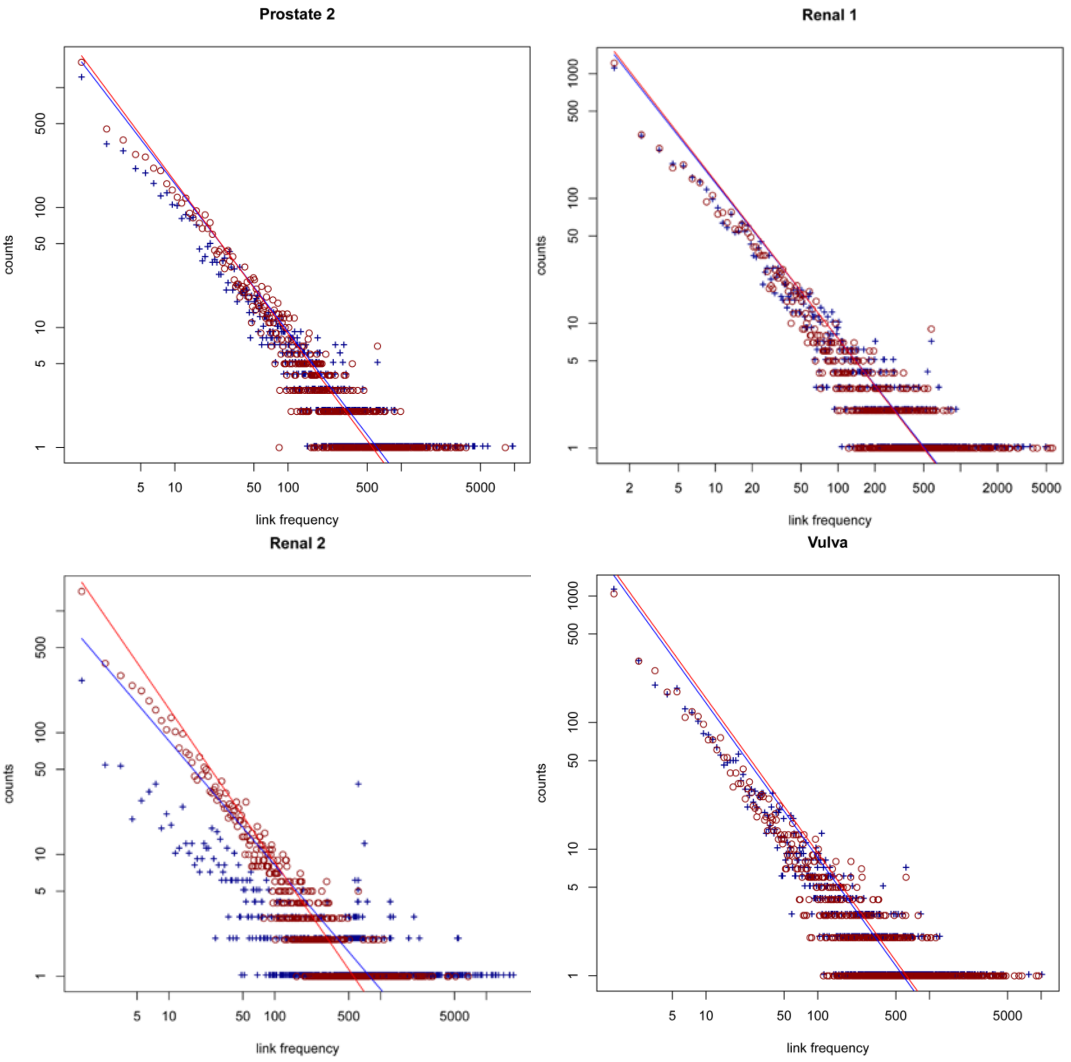

Figure S2. Link frequency distribution for all datasets (normal: blue crosses, cancer: red circles). All networks showed the typical scale-free distribution for the frequency of the genes to be involved in our defined signaling pathways. Most cancer networks exhibited a distinct steeper slope indicating less frequency for hubs in the network. All lines were fitted by a least squares fit (see methods).

y

Supplemental Figure S3. Visualization of the constructed networks

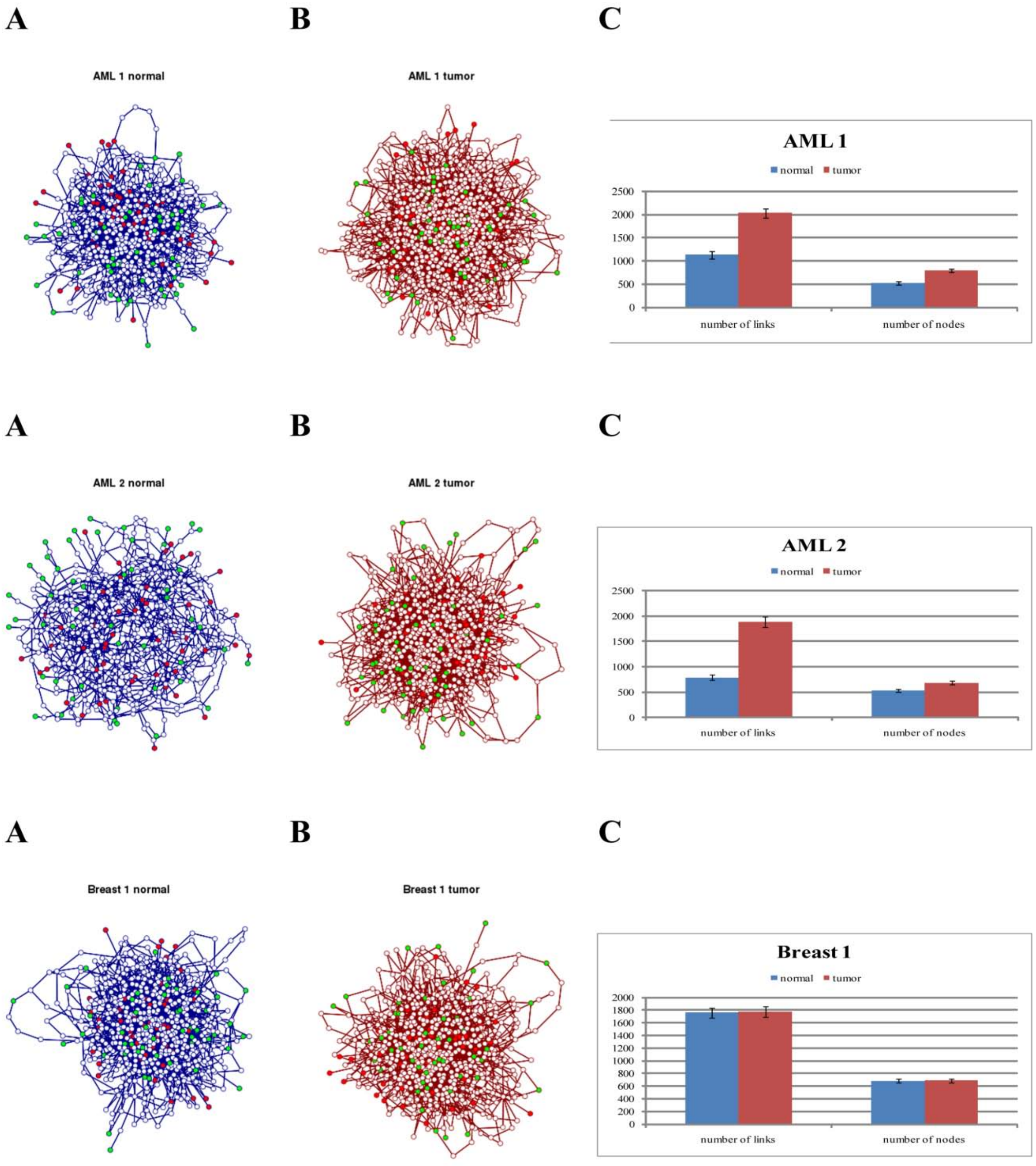

A

B

C

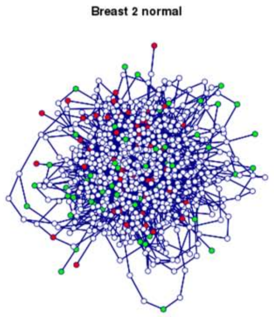

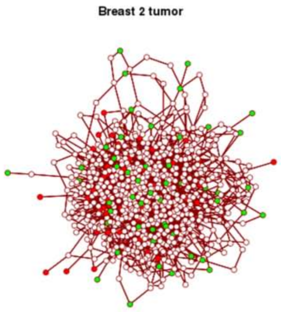

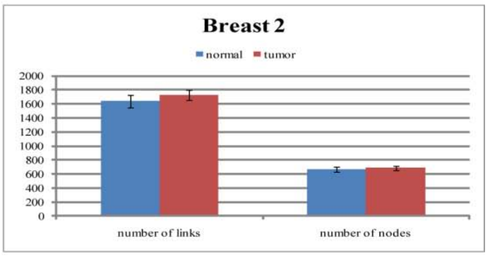

A

B

C

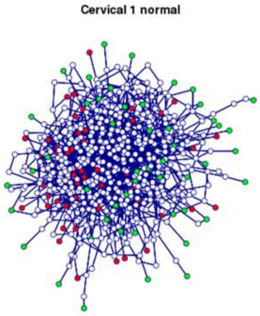

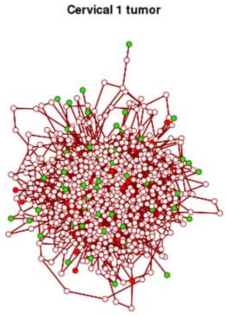

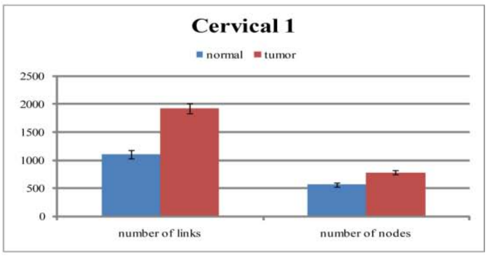

A

B

C

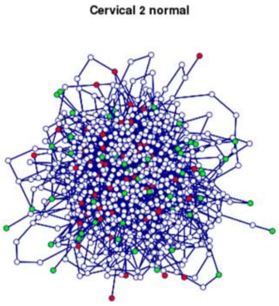

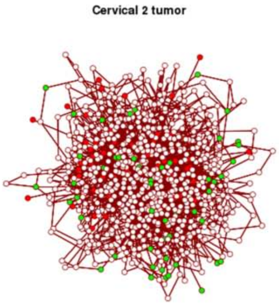

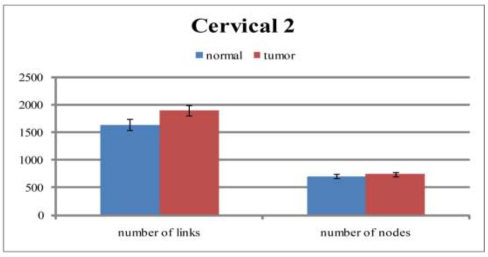

A

B

C

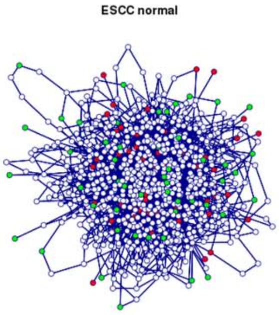

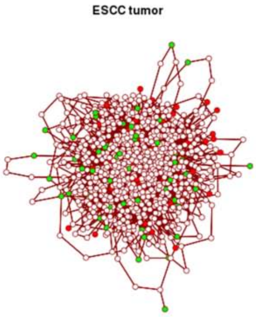

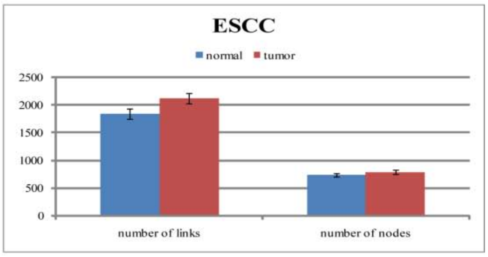

A B C

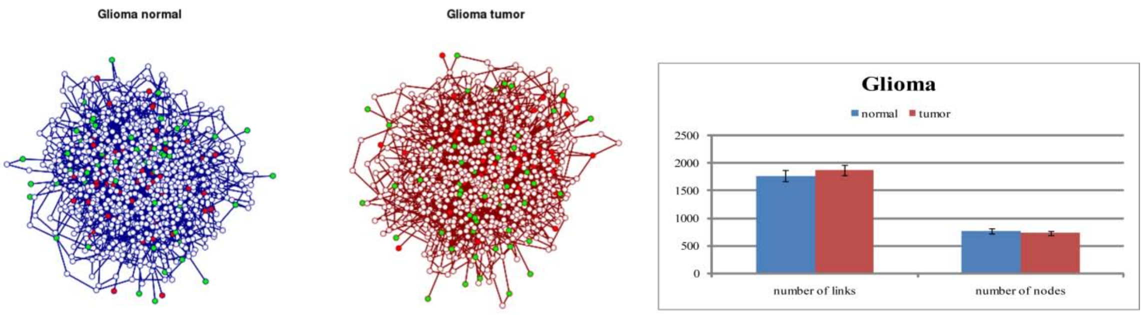

A B C

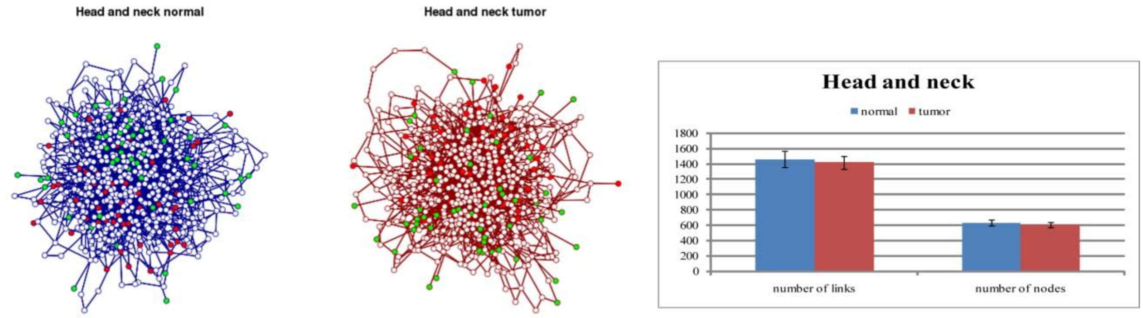

A B C

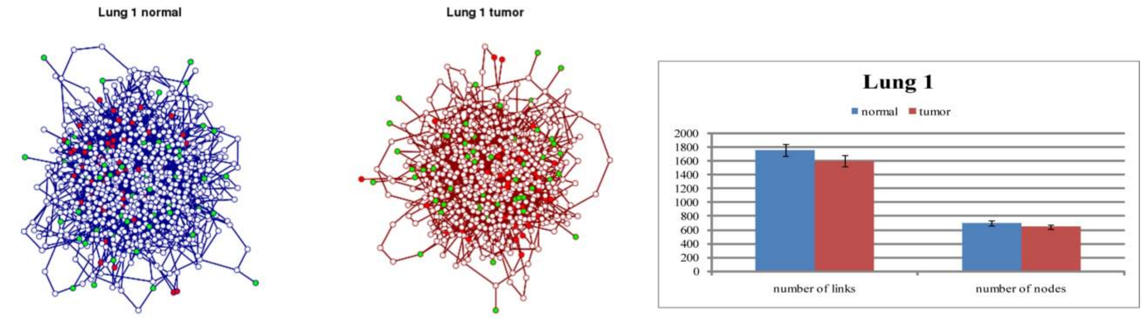

A B C

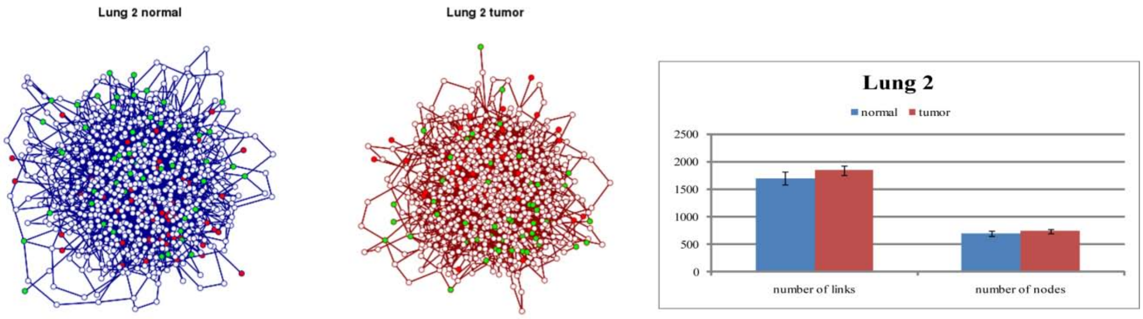

A B C

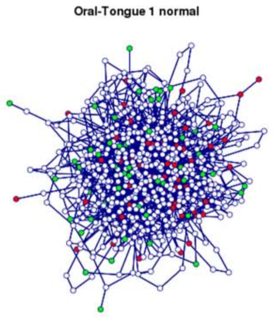

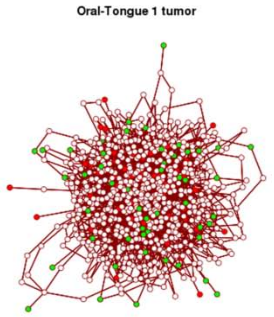

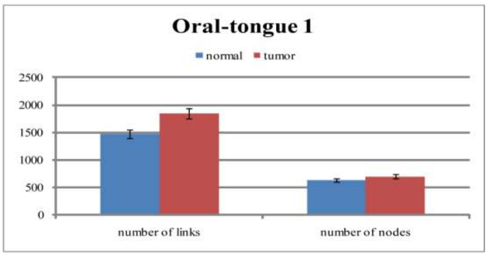

A B C

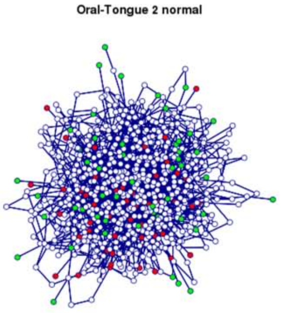

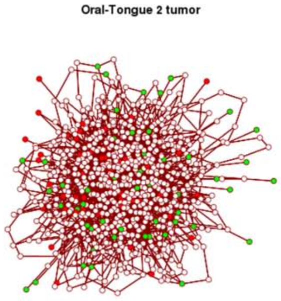

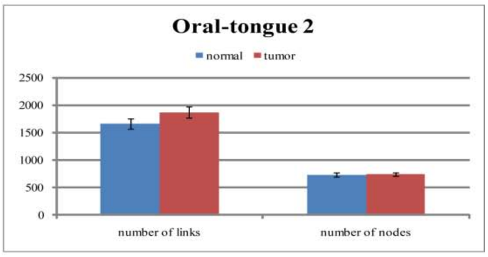

A B C

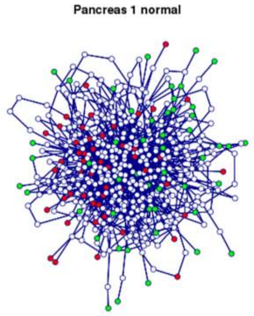

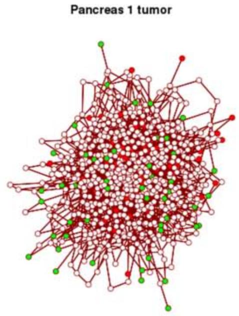

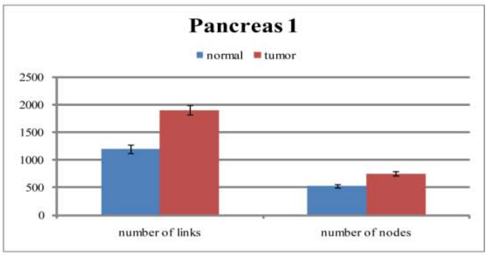

A B C

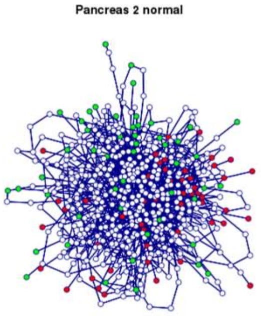

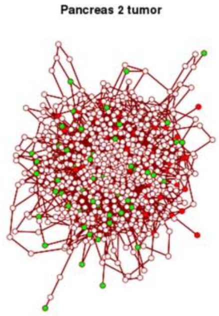

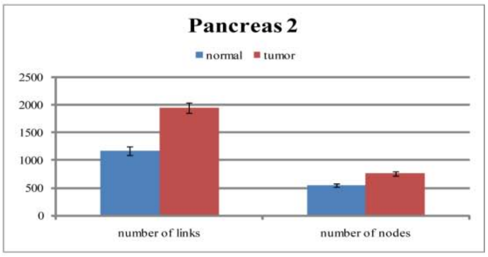

A B C

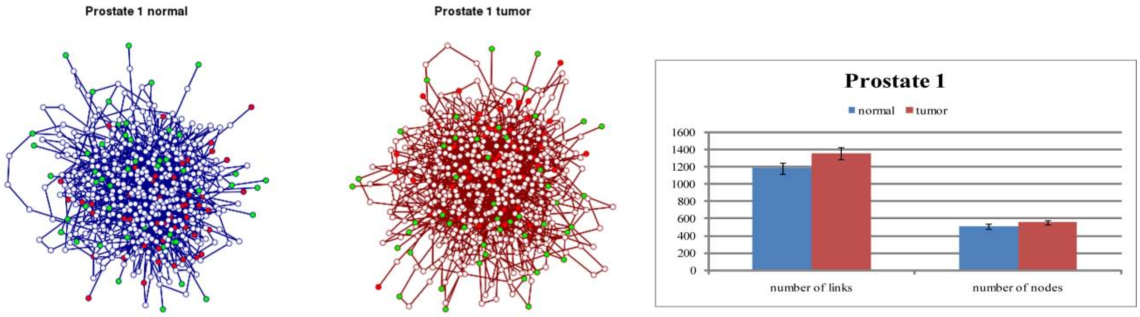
Prostate 1 normal
Prostate 1 tumor
Prostate 1
normal
tumor
1600
1400
1200
1000
800
600
400
200
0
number of links
number of nodes

A B C

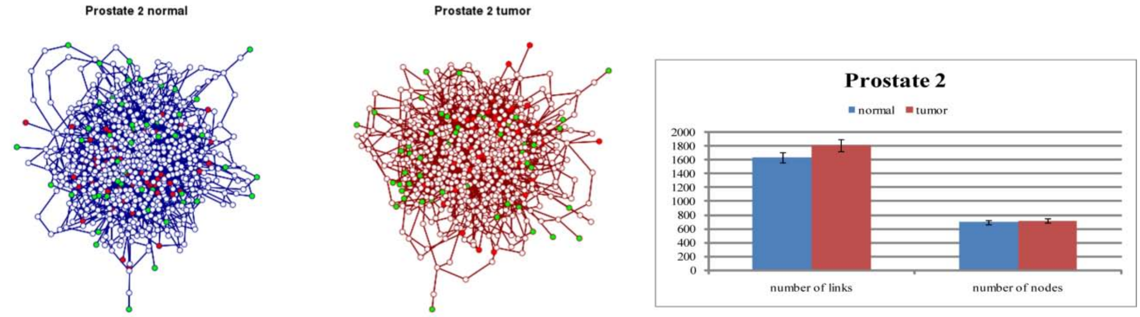
Prostate 2 normal
Prostate 2 tumor
Prostate 2
normal
tumor
2000
1800
1600
1400
1200
1000
800
600
400
200
0
number of links
number of nodes

A B C

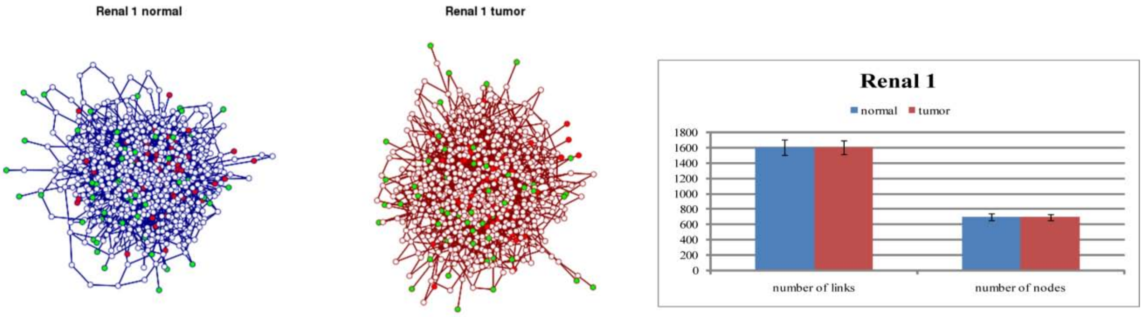
Renal 1 normal
Renal 1 tumor
Renal 1
normal
tumor
1800
1600
1400
1200
1000
800
600
400
200
0
number of links
number of nodes

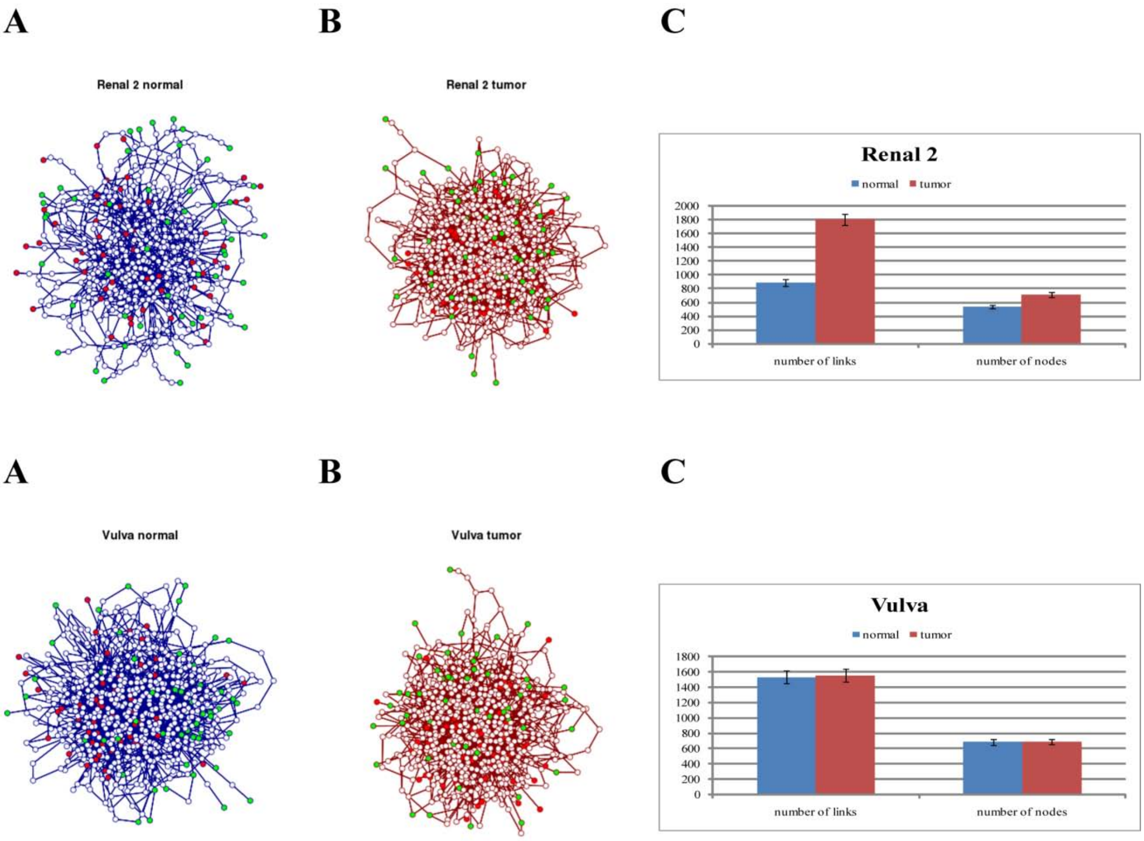

Figure S3. For visualization, we randomly selected 50 receptors (green nodes) and 50 transcription factors (red nodes) and constructed (A) the normal networks (blue links) and (B) cancer networks (red links) as described in the method section. (C) We repeated this procedure 50 times and calculated the mean number of links (normal: 1447.5, tumor: 1790.4, P = 1.7E-04) and the mean number of nodes (normal: 637.4, tumor: 710.2, P = 2.3E-03) for the networks of the specific cancer types (normal: blue, tumor: red bars) and found higher numbers in the cancer networks in good agreement with our findings. The error bars show the standard deviations (1σ) of 50 repetitions.

y
